# Conserved Amino Acids Residing Outside the Voltage Field Can Shift the Voltage Sensitivity and Increase the Signal Speed and Size of *Ciona* Based GEVIs

**DOI:** 10.3389/fcell.2022.868143

**Published:** 2022-06-16

**Authors:** Masoud Sepehri Rad, Lawrence B. Cohen, Bradley J. Baker

**Affiliations:** ^1^ Brain Science Institute, Korea Institute of Science and Technology (KIST), Seoul, South Korea; ^2^ Department of Neuroscience, University of Wisconsin, Madison, WI, United States; ^3^ Cellular and Molecular Physiology, Yale University School of Medicine, New Haven, CT, United States; ^4^ Division of Bio-Medical Science and Technology, KIST School, Korea University of Science and Technology (UST), Seoul, South Korea

**Keywords:** voltage, voltage imaging, GEVI, fluorescence, voltage sensing domain

## Abstract

To identify potential regions of the voltage-sensing domain that could shift the voltage sensitivity of *Ciona intestinalis* based Genetically Encoded Voltage Indicators (GEVIs), we aligned the amino acid sequences of voltage-gated sodium channels from different organisms. Conserved polar residues were identified at multiple transmembrane/loop junctions in the voltage sensing domain. Similar conservation of polar amino acids was found in the voltage-sensing domain of the voltage-sensing phosphatase gene family. These conserved residues were mutated to nonpolar or oppositely charged amino acids in a GEVI that utilizes the voltage sensing domain of the voltage sensing phosphatase from *Ciona* fused to the fluorescent protein, super ecliptic pHluorin (A227D). Different mutations shifted the voltage sensitivity to more positive or more negative membrane potentials. Double mutants were then created by selecting constructs that shifted the optical signal to a more physiologically relevant voltage range. Introduction of these mutations into previously developed GEVIs resulted in Plos6-v2 which improved the dynamic range to 40% ΔF/F/100 mV, a 25% increase over the parent, ArcLight. The onset time constant of Plos6-v2 is also 50% faster than ArcLight. Thus, Plos6-v2 appears to be the GEVI of choice.

## Introduction

Genetically encoded voltage indicators (GEVIs) are potentially powerful tools for monitoring electrical activity in the brain. Kinetics, brightness and the signal size are some of the important properties of a GEVI (Bando et al., 2019; Rhee et al., 2020). Optimizing the properties of the GEVIs is important for improving the utility of GEVIs for imaging fast electrical activities in neural tissue and *in vivo* (Storace et al., 2015; Inagaki et al., 2017). GEVIs with fast kinetics and large dynamic signals are needed to follow the voltage transients of neurons. In recent years, several attempts have been reported to improve GEVI’s properties (Gong et al., 2015; Piao et al., 2015; Lou et al., 2016; Storace et al., 2016; Jung et al., 2017; Lee et al., 2017; Sepehri Rad et al., 2017; Piatkevich et al., 2018; Adam et al., 2019; Lee et al., 2019).

The first GEVI to consistently yield voltage-dependent optical signals in mammalian cells was VSFP2 which consisted of two FPs fused in tandem to the carboxy terminus of the voltage sensing domain (VSD) from the *Ciona* voltage sensing phosphatase (Dimitrov et al., 2007). The two FPs were the Fluorescence Resonance Energy Transfer (FRET) donor/acceptor pair of Cerulean and Citruline enabling voltage induced conformational changes of the protein to alter the FRET efficiency thereby producing an optical signal. Given that FRET efficiency is dependent upon the distance and orientation of the FRET pair chromophores, the tandem fusion of the FPs limited the dynamic range of the GEVI’s response. Several approaches were employed to improve VSFP2. One approach was to separate the FRET pair with the VSD resulting in Nabi (Sung et al., 2015) and VSFP Butterfly (Akemann et al., 2012) having one FP near the N-terminus while the other was at the C-terminus. VSFP Butterfly has had some success in reporting neuronal activity *in vivo* (Akemann et al., 2012; Carandini et al., 2015; Empson et al., 2015).

Another approach was to systematically replace the FRET pair at the C-terminus with single FPs including the pH-sensitive Super Ecliptic pHluorin (Miesenbock et al., 1998; Ng et al., 2002) and screen for voltage sensitivity. In an effort to reduce the variable expression of these GEVIs during screening, stable cell lines were created which was to have a profound effect of GEVI development (Jin et al., 2012). All of the stable cell lines expressing the GEVI with Super Ecliptic pHluorin yielded a very small signal of around 1% ΔF/F/100 mV except for one. That one exception gave a 15% ΔF/F/100 mV due to a spontaneous mutation converting the alanine at position 227 in Super Ecliptic pHluorin to aspartic acid. Optimization of the linker length between the VSD and the FP domain resulted in the GEVI, ArcLight, which remains one of the best GEVIs available to date (Bando et al., 2019).

ArcLight yields a large change in fluorescence in response to changes in membrane potentials (Jin et al., 2012). ArcLight can give up to a 40% ΔF/F for a 100 mV depolarization of the plasma membrane in HEK 293 cells (Han et al., 2013). However, its fast time constant is ∼10 ms comprising 65% of the optical signal. Since action potentials are 1–2 ms, ArcLight will only reach ∼10% of its maximal signal by the time the spike has subsided. To improve ArcLight, mutagenesis on the *Ciona* phosphataseVSD was performed. Using the architecture of ArcLight with the wildtype *Ciona* VSD fused to Super Ecliptic pHluorin yielded the GEVI, CC1 (Piao et al., 2015). CC1 required strong depolarization of the plasma membrane (∼150 mV depolarization step) to observe a fluorescence change providing an excellent control for monitoring shifts in the voltage response to more negative potentials. Introducing mutations to conserved polar resides in the transmembrane segments of the VSD altered the voltage range, the speed of optical response, and the signal size of the GEVI (Piao et al., 2015). The resulting probe, Bongwoori, exhibited faster kinetics enabling the resolution of action potentials in a hippocampal neuron firing at 60 Hz. Altering the composition of the amino acid linker between the VSD and the FP domain of Bongwoori further improved the dynamic response resulting in two novel GEVIs, Bongwoori-Pos6 and Bongwoori-R3 that differ in their voltage sensitivities (Lee et al., 2017). The plasma membrane potential at which 50% of the total fluorescence change occurs (V_1/2_) was near -30 mV for Bongwoori-Pos6 while Bongwoori-R3 is near 0 mV. Bongwoori-Pos6 is potentially more suited for measuring subthreshold potentials while Bongwoori-R3 is more suited for action potentials.

**FIGURE 1:**
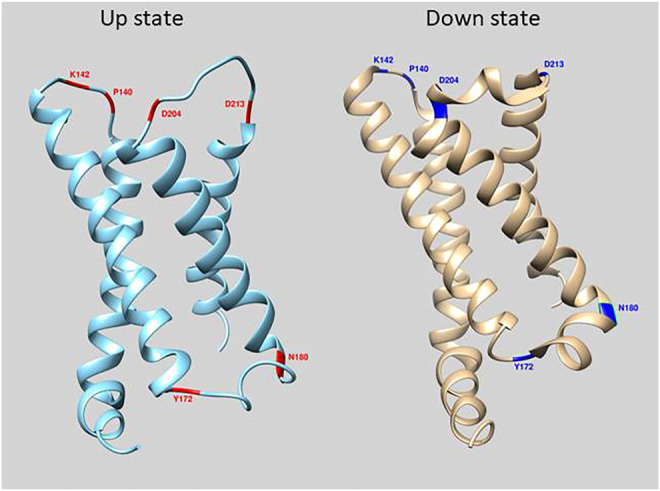
Location of mutations in loop regions of the VSD of Ciona Voltage Sensing Phosphatase. The crystal structure of the down state is in cyan and up state is in beige (Li et al., 2014). Red and Blue regions depict amino acid positions that were mutated in this study.

In this report, we investigate the effect on the voltage-dependent optical signal that amino acids in the cytosolic and extracellular loops in the voltage sensing domain (VSD) have for GEVIs based on the voltage sensing phosphatase gene from *Ciona* (Murata et al., 2005). Previously, we have shown that introducing mutations near the external transmembrane/loop junction of the first transmembrane segment (S1) of the VSD in ArcLight (Figure 1) altered the cellular expression pattern enabling the optical reporting of voltage changes in internal membranes (Sepehri Rad et al., 2018). However, this only occurred in about 20% of the cells expressing this mutated GEVI, Aahn. The other 80% expressing Aahn yielded optical signals dominated by the plasma membrane signals.

Here we expand the mutagenesis to other loop regions in the VSD and report the effect on the size and voltage range of the voltage dependent optical signal. Aligning the VSD from the *Ciona* VSP to different subtypes of voltage-gated sodium channels from several different organisms identified conserved polar amino acids near the transmembrane/loop junctions in the VSD. Mutagenesis of these conserved polar amino acids in intra/extracellular loops in the VSD affected the voltage range of the *Ciona* based GEVIs even though they reside outside of the voltage field. Making double mutant constructs, we have successfully shifted the voltage sensitivity of CC1 to more negative potentials and improved its signal size for physiologically relevant voltage ranges.

## Materials and Methods

### Plasmid DNA Designs and Construction

The CC1(WT) construct was described in Piao et al., 2015. Double mutant probes [CC1(WT)-D204K-Y172A and CC1(WT)-D204K-Y172S] were made by a two-step PCR process using CC1(WT)-Y172A and CC1(WT)-Y172S as template DNA respectively. Primers used for amplification of the S1, S2, and S3 transmembrane domains with a single mutation (D204K) in first step PCR reaction were: LC226: 5-ATA CGA CTC ACT ATA GGG-3 and LC230: 5- acc​GTA​TTC​CTT​TAA​CAC​AGT -3. Primers used for amplification of S4 transmembrane domain and florescence protein in first step PCR reaction were: LC229: 5- ACT GTG TTA AAG GAA TAC ggt -3 and LC193: 5- GCG​ATA​TCT​TCT​TTT​GTT​aaa​a -3. In the second step PCR, we used primers LC226 and LC193 and combined the first step PCR products. The second step PCR product then was digested with restriction enzymes Nhe1 and Kpn1 and inserted into the corresponding sites of the CC1(WT) construct. Similarly, we used CC1(WT)-Y172A and CC1(WT)-Y172S as template DNA respectively to generate the double mutant probes [CC1(WT)-D204A-Y172A and CC1(WT)-D204A-Y172S]. For amplification of the S1, S2, and S3 transmembrane domains with a single mutation (D204A) in first step PCR reaction these primers were used: LC226: 5-ATA CGA CTC ACT ATA GGG-3 and LC232: 5- acc​GTA​TTC​GGC​TAA​CAC​AGT -3. Primers used for amplification of S4 transmembrane domain and florescence protein in first step PCR reaction were: LC231: 5- ACT GTG TTA GCC GAA TAC ggt -3 and LC193: 5- GCG​ATA​TCT​TCT​TTT​GTT​aaa​a -3. We then used primers LC226 and LC193 to combine the first step PCR products. Using enzymes Nhe1 and Kpn1, the second step PCR product then was digested and inserted into the corresponding sites of the CC1(WT) construct. All DNA constructs were confirmed by DNA sequencing (Cosmogenetech, Republic of Korea).

### Cell Culture

HEK293 cells were maintained in DMEM (High Glucose DMEM; Gibco) supplemented with 10% (v/v) fetal bovine serum (FBS; Invitrogen). HEK293 cells were seeded on to #0 coverslips coated with poly-l-lysine (Ted Pella, Inc.) in a 24-well culture dish and kept in an incubator at 37°C under air with 5% CO_2_. Transfection was performed by using Lipofectamine 2000 (Invitrogen) following the manufacturer’s instructions. Cells were roughly 20% confluent when transfected and were imaged 24 to hours after transfection.

### Patch Clamp

Electrophysiology recordings were performed at 33°C and the chamber was perfused with a bath solution containing 150 mM NaCl, 4 mM KCl, 2 mM CaCl2, 1 mM MgCl2, 5 mM d-Glucose, and 5 mM HEPES (pH 7.4). Glass patch pipettes (capillary tubing with 1.5/0.84 mm; World Precision Instruments) were pulled by a P-97 micropipette puller (Sutter Instruments, United States) to make patch pipettes with 3–5 MΩ resistance when filled with internal solution containing (in mM) 120 K-aspartate, four NaCl, four MgCl_2_, one CaCl_2_, 10 EGTA, three Na2ATP, and five HEPES, pH 7.2. Using a Patch Clamp EPC10 amplifier (HEKA) with a holding potential of −70 mV, whole-cell voltage clamp was done in transfected HEK293 cells. The osmolality of the bath solution was 318 mOsm/kg and that of the pipette solution was 291 mOsm/kg H_2_O.

### Wide-Field Imaging

We used an inverted microscope (IX71; Olympus, Japan) equipped with a 60X oil-immersion lens with 1.35-numerical aperture (NA) for imaging the whole-cell patch clamped cells. Illumination light was provided by a 75 W Xenon arc lamp (Cairn Research). The excitation filter for all constructs was 472/30 nm, the emission filter was 496/LP and the dichroic was 495 nm (Semrock, NY). The fluorescence image was demagnified by an Optem zoom system, A45699 (Qioptiq LINOS) and the sample imaged onto a NeuroCCD-SM camera with 80 × 80 pixels controlled by NeuroPlex software (RedShirtImaging, GA). The images were recorded at a frame rate of 500 fps.

### Optical Signal Analysis

Acquired images from patch clamp fluorometry were analyzed using NeuroPlex software (RedShirtImaging, United States), Excel (Microsoft, United States), and Origin8.6 (Origin Labs, United States). The resulting traces from whole cell voltage clamp experiments of HEK 293 cells were averaged for 16 trials. To calculate the % ΔF/F, we first subtracted the dark image from all frames, then the average of a region of interest in each frame during a voltage step (F) was subtracted from the average of the region taken from ten frames prior to the event of interest (F0), and finally this value was divided by F0 by the following formula: %∆F⁄F=[(F-F_0)/F_0]100. ΔF/F values for all of the tested constructs were plotted in OriginPro 2019 (OriginLab, United States) and fitted to a Boltzmann function (signal size was normalized from zero, minimum, to one, maximum) to acquire voltage sensitivities as previously described (Piao et al., 2015). Frame subtraction images were achieved by subtracting the average of light intensities of 20 frames during the 200 mV depolarization step from average of 20 frames at the beginning of the recording.

## Results

The GEVI, CC1, contains the wild type VSD sequences of the *Ciona intestinalis* voltage sensing phosphatase gene with the FP, Super Ecliptic pHlorin A227D, fused to the carboxy-terminus of the protein. Introduction of mutations in the α-helix/loop junctions in the VSD of CC1 yielded probes with shifted voltage sensitivity in a more positive or a more negative direction. To identify other regions of the VSD which contribute to determining the voltage sensitivity, 148 voltage-gated sodium channels (Nav) from different organisms were aligned with the VSD from Ciona VSP. Nav channels were chosen since they have four VSDs in each protein. The four VSDs from each sodium channel were separated into distinct sequences. The resulting alignment consisted of 593 VSDs (Supplementary Figure S1). Conserved polar residues were detected at multiple transmembrane/loop junctions (Figure 1), two sites in the S1-S2 loop (I140 and K142), two sites in the S2-S3 loop (Y172 and N180), and another two sites in the S3-S4 loop of the VSD (D204 and D213). These conserved residues were mutated to nonpolar or oppositely charged amino acids.

### S1-S2 Loop Mutants

**FIGURE 2:**
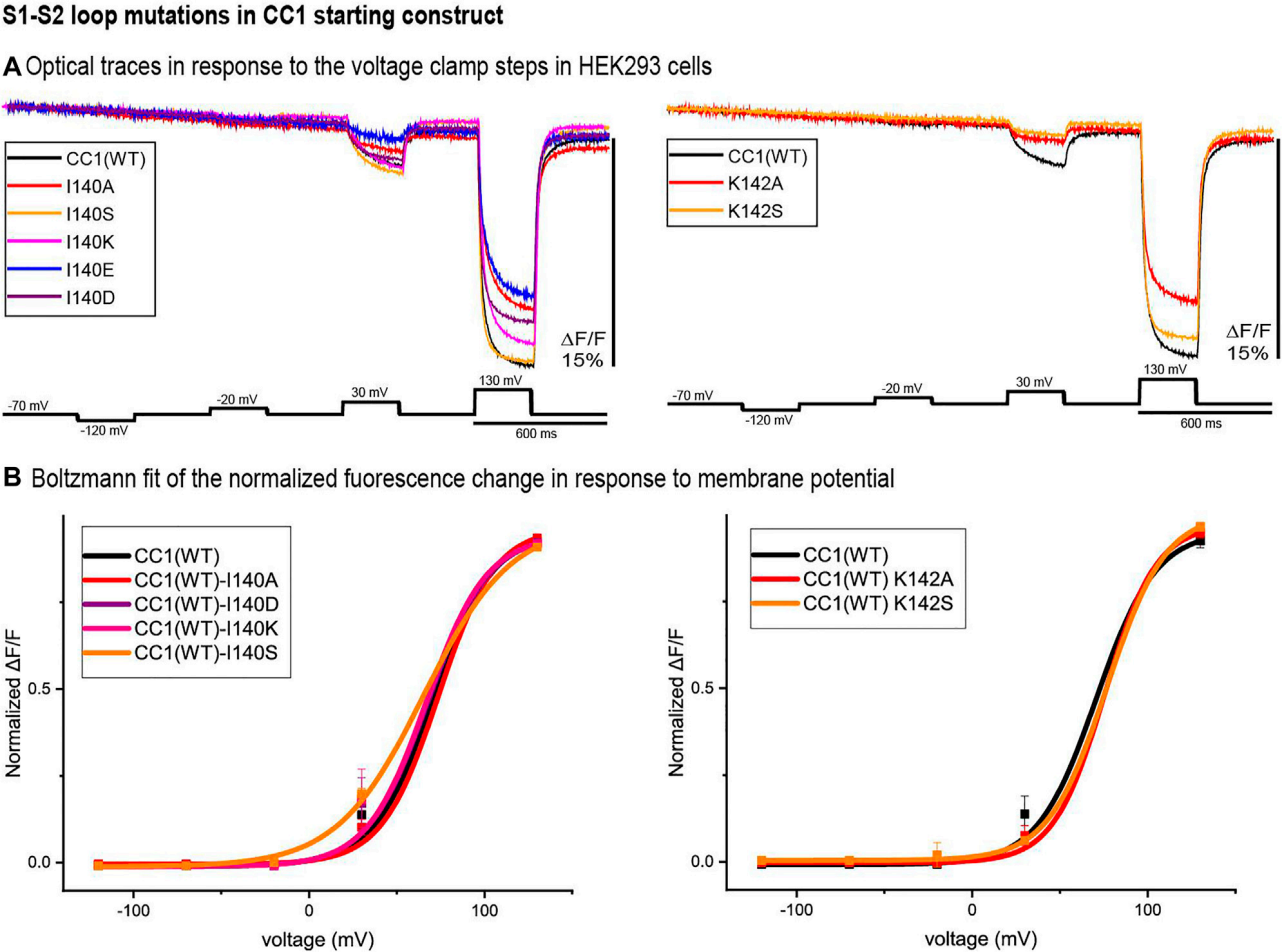
S1-S2 loop mutations in the CC1 starting construct. **(A)**. The traces are representative fluorescent signals from voltage-clamped HEK293 cells (average of 16 trials for each cell) expressing CC1(WT) or CC1 with an S1-S2 loop mutant. Each trace is the average of three cells. The holding potential was −70mV. The pulse protocol is given in the black trace and was identical for all constructs. No temporal filtering was used for the traces. Images were recorded at a frame rate of 500 fps. **(B)**. Boltzmann fit of the normalized fluorescence change in response to membrane potential (*n* = 3 for all constructs).

Two potential sites of interest were identified in the S1-S2 loop, I140 and K142. Figure 2 compares the traces of the S1-S2 loop mutations at those locations with that of the original CC1 construct. Except for I140S, none of the S1-S2 loop mutations shifted the voltage sensitivity significantly. While the I140A, I140E, K142A, and K142S mutations decreased the signal size for a 100 mV depolarization, I140S increased the signal size from 2.5 to 3.5% (Figure 2A). The I140S mutation shifted the V_1/2_ voltage at half-maximum signal from +70 mV to +48 mV (Figure 2B).

### S2-S3 Loop Mutants

**FIGURE 3:**
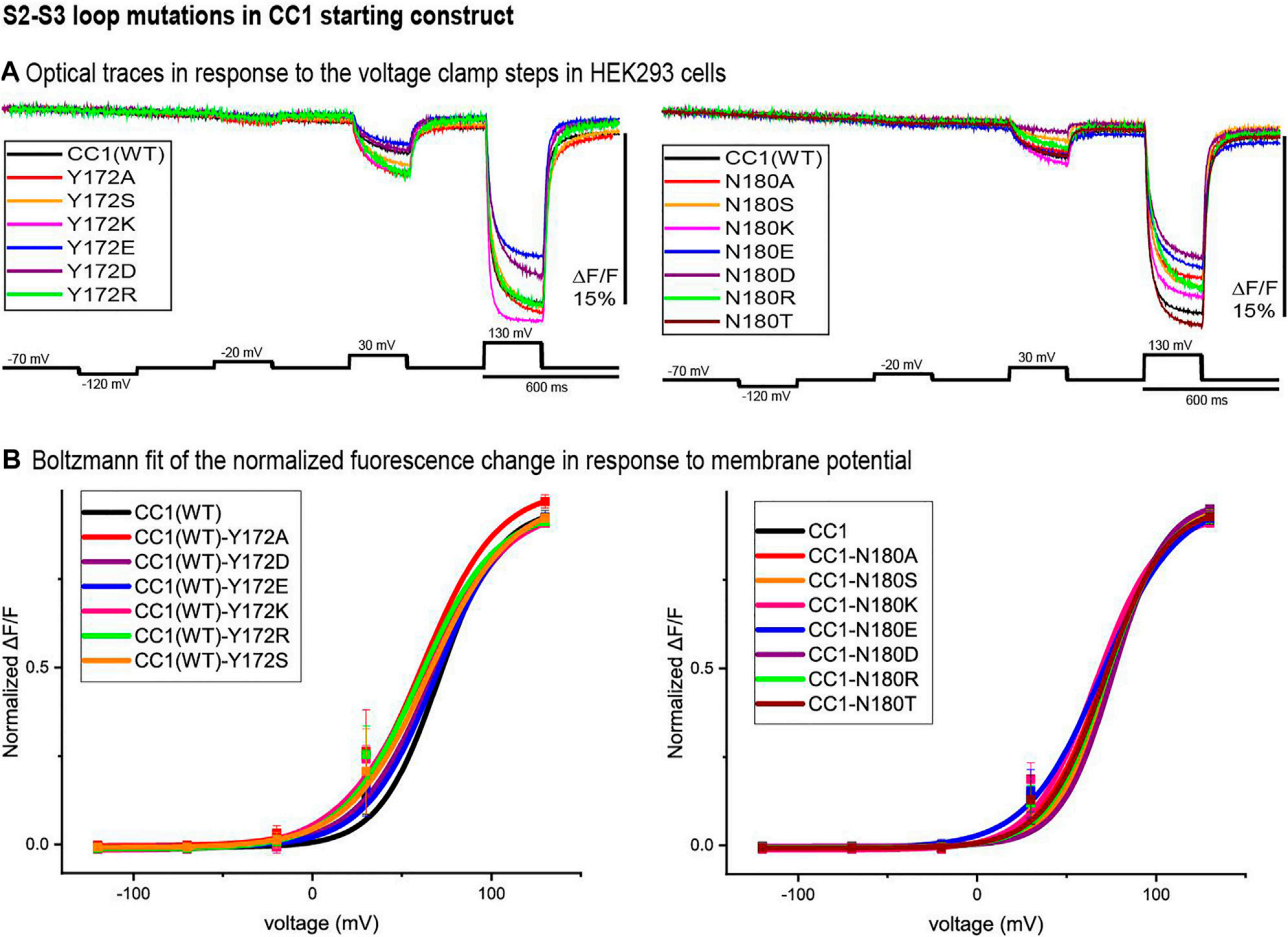
S2-S3 loop mutations in the CC1 starting construct. **(A)**. Representative fluorescent traces of HEK cells expressing the CC1(WT) or CC1(WT) with an S2-S3 loop mutant in the whole cell patch clamp configuration. The cells were subjected to the voltage command pulses in black. Each trace is from average of three cells and the results from each cell is the average of 16 trials. The traces are fluorescent optical signals from plasma membrane without temporal filtering. Images were recorded at a frame rate of 500 fps. **(B)**. Boltzmann fit of the normalized fluorescence change for the CC1 construct and its derivative probes with single mutation in the S2-S3 loop (*n* = 3 for all constructs).

Figure 3 illustrates the optical signal traces of the intracellular S2-S3 loop mutants in comparison to the CC1 starting construct. While Y172D did not change the signal size for a 100 mV depolarization, Y172A, Y172S, Y172K, and Y172R did increase the signal size compared to the CC1. Y172R and Y172K increased the signal size from 2.5 to 4.8% and 5% respectively. In contrast, the N180D mutation had a deleterious effect decreasing the signal size from 2.5 to 0.8% for a 100 mV step (Figure 3A). The Y172 mutations tested slightly shifted the voltage response to more negative potentials, with Y172A having the largest effect. This single mutation shifted the voltage at half maximum from +70 mV to +45 mV. The mutations in the S2-S3 loop junction (N180) did not change the voltage sensitivity significantly (Figure 3B).

### S3-S4 Loop Mutants

**FIGURE 4:**
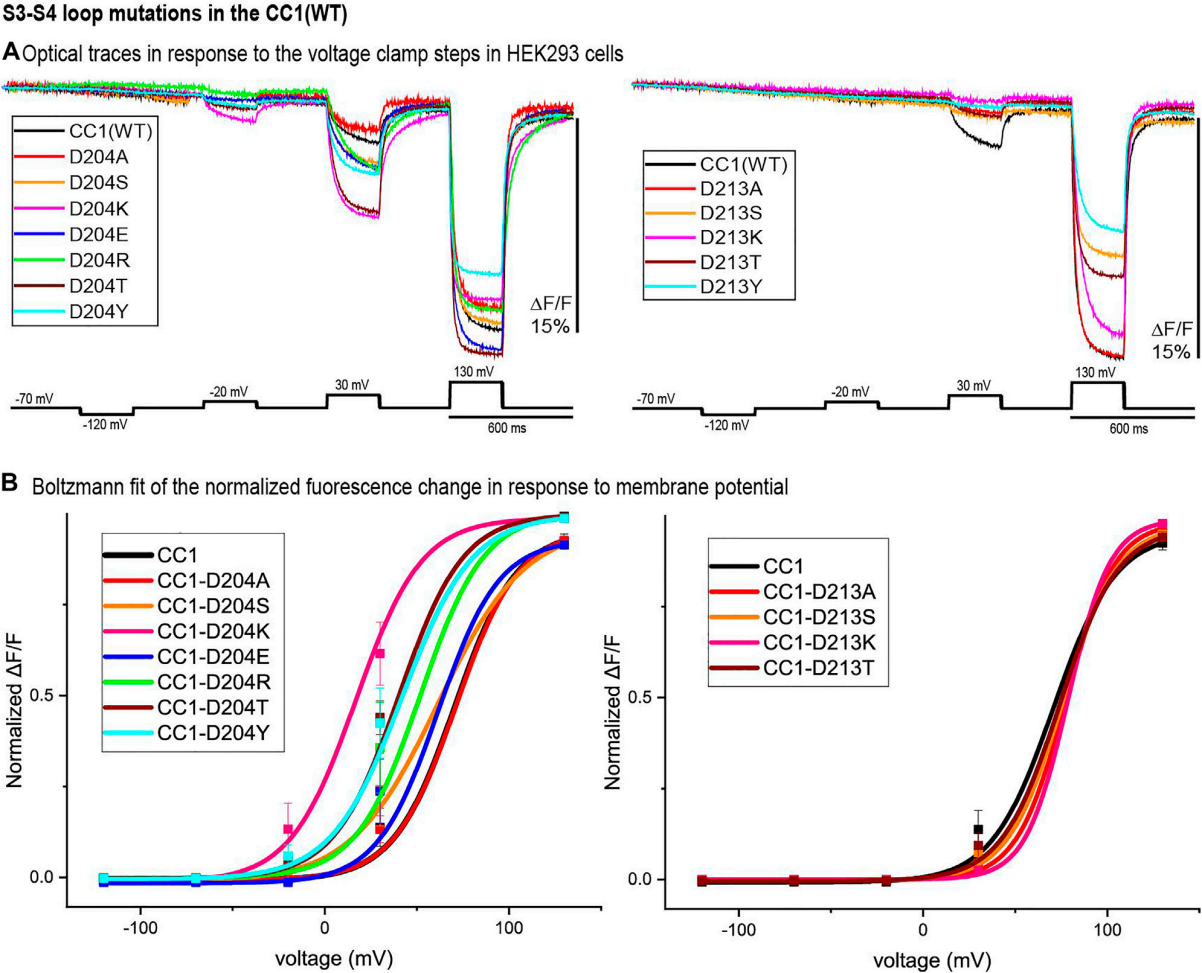
S3-S4 loop mutations in the CC1 starting construct. **(A)**. HEK 293 cells expressing the CC1(WT) or CC1(WT) with an S3-S4 loop mutant were voltage-clamped. The traces are fluorescent optical signals (average of three cells) from plasma membrane without temporal filtering. Each cell is the average of 16 trials. Images were recorded at a frame rate of 500 fps. **(B)**. The voltage-fluorescence curve of the normalized optical signal for the CC1 construct and its derivative probes with single mutation at S3-S4 loop (*n* = 3 for all constructs).

Figure 4 represents the optical signal traces from the extracellular S3-S4 loop mutants. D204S, D204K, D204E, D204R, D204T, and D204Y have a larger signal size for 100 mV depolarizations compared to the parent CC1 signal; D204K has the largest effect increasing the signal size from 2.5 to 8.1% for a 100 mV depolarization to +30 mV. D213A, D213S, D213K, D213T, D213Y, and D204A have a smaller signal size for 100 mV depolarizations (Figure 4A). While the D204A mutation did not shift the voltage versus fluorescence trace, all of the other mutations tested at this location shifted the voltage response of the CC1 probe to more negative potentials. D204K had the most pronounced effect shifting the V_1/2_ from +70 mV to +17 mV. Mutations at the D213 position were mostly deleterious with none of the new constructs yielding a signal larger than the original CC1 version (Figure 4B).

### Double Mutants

**FIGURE 5:**
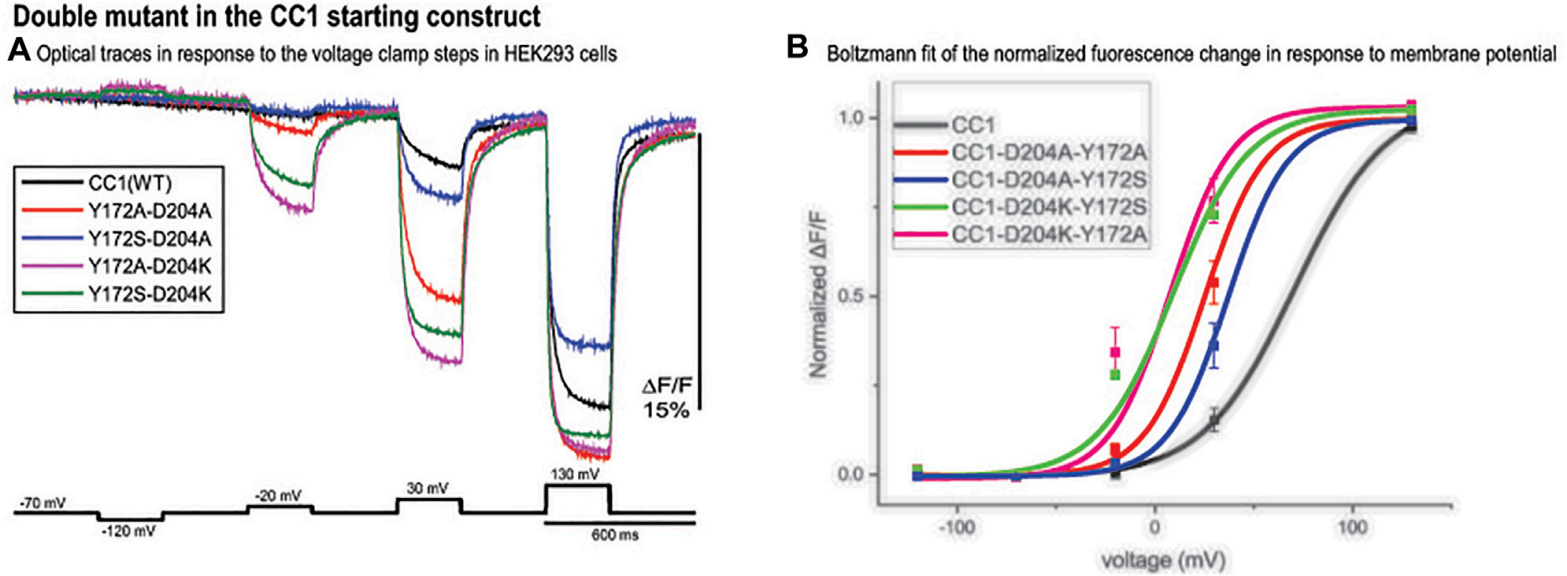
Double mutant in the CC1 starting construct. **(A)**. Fluorescence response of HEK293 cells expressing CC1(WT) or CC1(WT) with double mutations in the loops to depolarizing and hyperpolarizing voltage steps. The traces are the average of three cells (each cell is the average of 16 trials) without temporal filtering. Images were recorded at a frame rate of 500 fps. **(B)**. The optical signal was normalized and fitted to a Boltzmann equation. All double mutant combinations shifted the voltage response to more negative potentials (*n* = 3 for all constructs).

The CC1 starting construct has a voltage response shifted to more positive potentials compared to the physiological voltage range (-100 mV to +30 mV). We tried to further increase the signal size for a 100 mv depolarization by shifting the voltage sensitivity to even more negative potentials using double mutations. Comparing optical traces of the loop mutants with that of the starting CC1 construct we selected constructs that showed relatively large voltage shifts to more negative potentials and made four new probes. The voltage response of these double mutant constructs are shifted to even more negative potentials and as a result they give a larger signal compared to CC1 for a 100 mv depolarization (Figure 5A). Y172A/D204K had the largest effect (Figure 5B). This construct increased the signal size for 100 mv depolarizations compared to the CC1 optical signal from 2.5 to 13.8% for a 100 mv depolarization.

### A Triple Mutant

**FIGURE 6:**
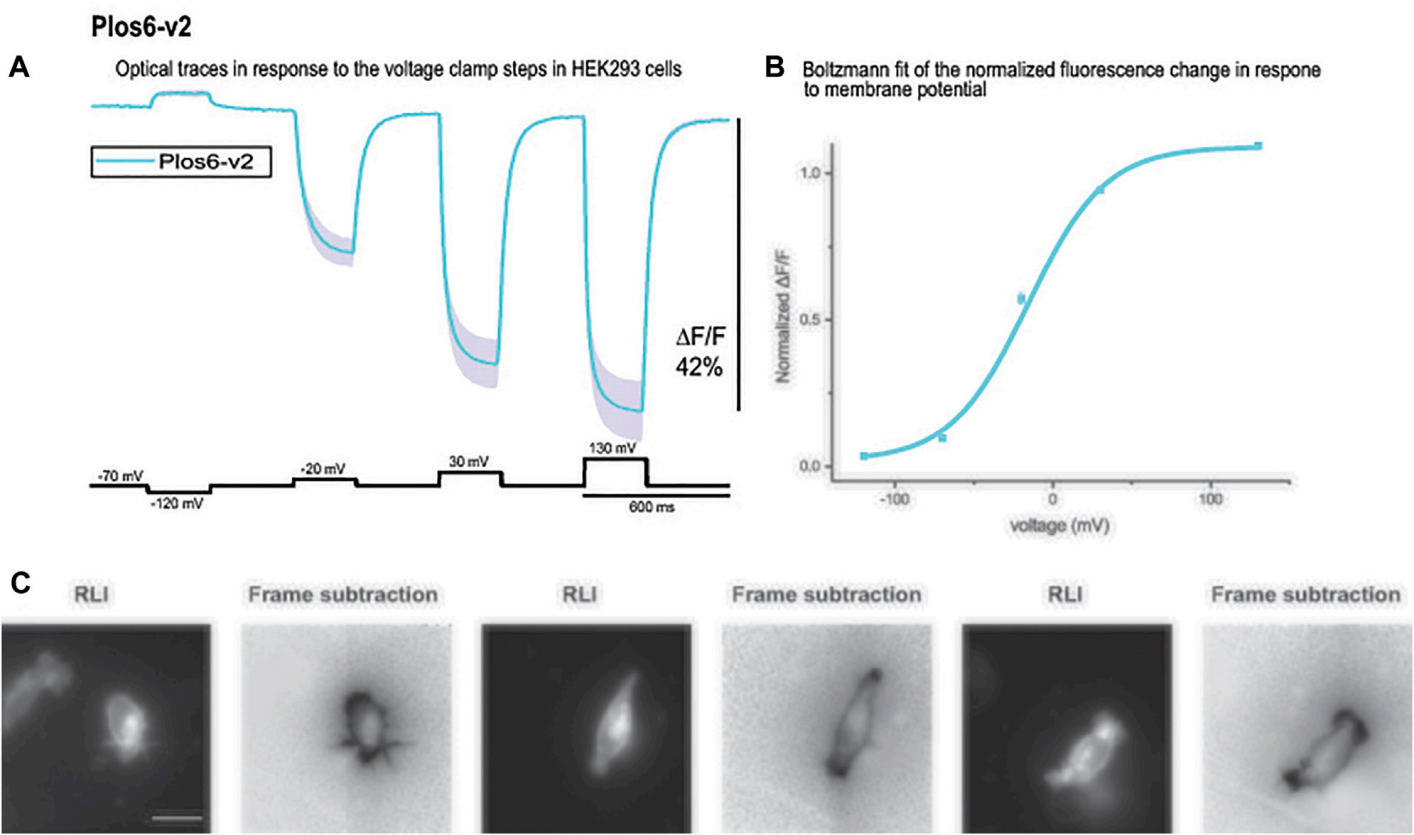
The large fluorescence response of Plos6-v2. **(A)**. Plos6-v2 was expressed in HEK-293 cells and subjected to whole cell voltage clamp. The dark line represents the average of three cells. The shaded region is the standard error of the mean. **(B)**. Plos6-v2 Blotzmann fit of the fluorescence change as a function of voltage. **(C)**. Resting light images (RLI) and corresponding frame subtraction images of three cells expressing Plos6-v2. The fluorescence average for each pixel from 20 frames during the 200 mV depolarization step were subtracted from the fluorescence average of 20 frames at the beginning of the recording when the cells were held at a holding potential of −70 mV. The scale bar represents 20 μm.

Bongwoori-Pos6 is an ArcLight derived-GEVI with a modified linker region connecting the VSD to the FP domains of the GEVI (Lee et al., 2017). The V_1/2_ for Bongwoori-Pos6 is -28 mV and exhibits an 18% ΔF/F/100 mV depolarization step of the plasma membrane. Bongwoori-Pos6 also exhibits faster kinetics with a *t* on of 6 ± 1 msec and a τ off of 8 ± 1 msec. Introducing the combination of the loop mutations that shifted the voltage response of CC1 (Y172A and D204K) with another mutation in the transmembrane segment S2 of the VSD (D164N) that has also been shown to affect the voltage range (Piao et al., 2015) with the Bongwoori-Pos6 linker resulted in a much improved signal size (Figure 6A). This new GEVI is designated Plos6-v2 and has a ∼40% for 100 mV depolarization step compared to the 18% signal for a 100 mV depolarization for Bongwoori-Pos6 (Lee et al., 2017). Plos6-v2 is also larger than the CC1 loop mutants in Figure 5. D204K-Y172A is 34.1%; D204K-Y172K is 33.2%; D204K-Y172R is 29.0%; and D204K-Y172S is 35.8%.

The onset of the voltage-dependent signal for Plos6-v2 was best fitted by a double exponential decay yielding a fast time constant (τ on) of 5.8 ± 0.3 msec (*N* = 3) for the 50 mV depolarization step. However, the fast component only accounted for 25 ± 0.8% of the amplitude. The slow component was 24.8 ± 1.4 msec. The speed of the onset signal for the 100 mV depolarization step again exhibited a fast component near 6 msec (5.5 ± 0.1 msec), but the fast component improved to 63 ± 2.5% of the amplitude. The slow component for the 100 mV onset of the signal was 22.2 ± 0.1 msec. At 200 mV, the fast component of the onset signal was 4 ± 0.8 msec and constituted 69 ± 1.6% of the signal. The slow component for the 200 mV onset signal was 20.6 ± 1.6 msec. The speed of the off signal was best fitted by a single exponential decay function yielding a τ off of 15.4 ± 0.5 msec for the 50 mV depolarization step, 14.7 ± 0.6 msec for the 100 mV depolarization step, and 13.9 ± 0.5 msec for the 200 mV step.

As can be seen from the Boltzmann fit in Figure 6B, the voltage response of Plos6-v2 was more attuned to the physiological range of neuronal activities. Plos6-v2 exhibited good membrane expression with some discernable internal fluorescence. Plotting the frame subtraction difference of the light levels during the 200 mV depolarization step from the initial fluorescence observed at the holding nicely reveals the population of probe at the plasma membrane (Figure 6C). Regions of interest were then determined by the frame subtraction image.

## Discussion

The challenges of *in vivo* voltage imaging can be reduced by increasing the fluorescence change in response to voltage transients of a GEVI. Classical approaches to achieve a larger optical signal involve improving the trafficking of the protein and the speed of the optical response. The improved trafficking of a GEVI increases the population of probe that can respond to voltage transients as well as reduces the non-responding internal fluorescence thereby improving the signal-to-noise ratio (Rhee et al., 2020). Improving the speed of the GEVI can also improve the signal-to-noise ratio. For example, ArcLight exhibits a maximal 40% ΔF/F/100 mV depolarization step of the plasma membrane. The kinetics of that fluorescence change is best fit by a double exponential decay with a fast component (τ on ∼10 msec) accounting for 65% of the amplitude (Jin et al., 2012). As a result, a neuron expressing ArcLight would exhibit a nearly 5% change in fluorescence during the firing of a 2 msec action potential. Increasing the fast component of the voltage-induced optical signal to a τ on ∼ 2 msec would improve the change in fluorescence to 16% (Yi et al., 2018).

Another approach is to optimize the voltage-dependent optical signal of a GEVI by manipulating the voltage range of the GEVI (Jung et al., 2017). CC1-Pos6 gave a voltage-dependent optical signal of over 50% ΔF/F for a 200 mV depolarization of the plasma membrane (Lee et al., 2017). However, the signal size was below 10% ΔF/F/100 mV limiting the usefulness for imaging neurological activities. Mutagenesis of conserved polar amino acids in the transmembrane segments of the VSD affected the voltage sensitivity of Ciona based GEVIs (Piao et al., 2015) prompting several approaches to shift the voltage response of CC1-Pos6 while trying to maintain the large signal size Partial success was achieved with Bongwoori-Pos6 which had nearly a 20% ΔF/F/100 mV signal, improved kinetics, and a V_1/2_ near -30 mV.

Guided by the sequences of voltage-gated sodium channels, we demonstrate in this report that polar residues in the transmembrane loop junctions of the VSD can also affect the characteristics of the voltage-dependent optical signal. We identified several mutations in the helix/loop junctions in the VSD that shift the voltage sensitivity of the GEVI. In the S1-S2 loop region I140A, E and K142A, S mutations shifted the voltage sensitivity to more positive voltages while the I140S, K and D resulted in little change in the voltage range of the optical response. In the intracellular S2-S3 loop, Y172A, S, K and R mutations shifted the voltage response to more negative potentials and N180S, R, T shifted the voltage response to more positive potentials. Mutagenesis of D204S, K, E, R, T, and Y in S3-S4 loop shifted the voltage response to more negative potentials, while D204A and D213A, S, K, T, and Y mutations shifted the voltage response of the optical signal to more positive potentials.

Selecting mutations that showed the largest voltage shift to more negative potentials, we made four double mutant probes. The voltage response of these new GEVIs are shifted to even more negative potentials and as a result they gave a larger signal compared to CC1 for a 100 mv depolarization step (Figure 5A). Y172A/D204K showed the biggest effect, it increased the signal size from 2.5 to 13.8%. Combining the D164N/Y172A/D204K mutations with Bongwoori-Pos6 increased its optical signal size even further. The resulting construct, Plos6-v2, gives a 40% ΔF/F optical signal for a 100 mV depolarization (Figure 6A) which is a 10-fold improvement over the original CC1 GEVI (Piao et al., 2015) and a ∼2-fold improvement over Bongwoori-Pos6 (Lee et al., 2017). The speed of Plos6-v2 is comparable to the improved speed of Bongwoori-Pos6, an important result given the slow kinetics of previous GEVIs utilizing the D164N mutation in the S2 domain of the VSD (Piao et al., 2015). The V_1/2_ of Plos6-v2 is -22 mV (Figure 6B) which is well positioned for detecting action potentials as well as subthreshold synaptic activity.

ArcLight-derived GEVIs convert a voltage-induced conformational change of the protein into an optical signal allowing the visualization of neuronal circuit activity (Borden et al., 2017; Platisa et al., 2022). This mechanism involves the movement of the VSD (Siegel and Isacoff, 1997; Dimitrov et al., 2007; Villalba-Galea et al., 2009), the photophysical properties of the FP domain (Kang and Baker, 2016; Kang et al., 2021), the linker region connecting the VSD to the FP (Jung et al., 2015; Lee et al., 2017) as well as potential interactions between neighboring GEVIs via the dimerization of the VSD (Rayaprolu et al., 2018; Leong et al., 2021). By exploring the ability to alter the voltage range of a protein by mutating amino acids in the loop regions of the VSD, we have developed a novel GEVI with ∼40% ΔF/F/100 mV. While ArcLight has been reported to yield a 40% ΔF/F/100 mV, in our hands we consistently observe a 30% signal (Piao et al., 2015). The 40% signal of Plos6-v2 is a substantial improvement in the dynamic range and exbits faster kinetics than ArcLight. Plos6-v2 should therefore be a useful tool for optically monitoring neuronal activity.

## Data Availability

The raw data supporting the conclusion of this article will be made available by the authors, without undue reservation.
