# Supplementary material for: Conserved Amino Acids Residing Outside the Voltage Field Can Shift the Voltage Sensitivity and Increase the Signal Speed and Size of Ciona Based GEVIs
Source: Front Cell Dev Biol. 2022 Jun 16;10:868143. doi: 10.3389/fcell.2022.868143 (PMC9243531; doi:10.3389/fcell.2022.868143)
Supplement: Supplementary file 1 [file DataSheet1.docx]

Supplementary Material

# Supplementary Data

|  | S1 S2 |
| --- | --- |
|  | * * * |
| NP_001028998.1 VSP *Ciona intestinalis* | FGVFLIF---L-DIILMIIDLSL--PGKSESSQSFYDGMALALSCYFMLDLGLRIFAYGP |
| XP_003766750.1 Na_v_1.7 III *Sarcophilus harrisii* | FEMFMLLIILLSCATLTFEDINF--QKRKT-IMIILEYADKIFTYLFILEMLLKWMACGC |
| XP_008136835.1 Na_x_ III *Eptesicus fuscus* | FKCFIGLVTLLSTVALAFEDIYI--NQRKT-IKILLEYADMIFTYIFILEMLLKWVAYGF |
| XP_005315754.1 Na_x_ III *I. tridecemlineatus* | FRCFIGLVTLLSTGALAFEDIYI--DQRKT-IKILLEYADMIFTYIFILEMLLKWMAYGF |
| XP_010350732.1 Na_x_ III *S. bol. boliviensis* | FKCFIGLVTLLSTSTLTLEDIYI--DQRKT-VKILLEYADMIFTYIFILEMLLKWMAYGF |
| XP_010363599.1 Na_x_ III *Rhinopithecus roxellana* | FKCFIGLVTLLSAGTLAFEDIYI--DQRKT-IKILLEYADMIFTYIFILEMLLKWMAYGF |
| XP_002812597.1 Na_x_ III *Pongo abelii* | FKCFIGLVTLLSTSTLAFEDIYI--DQRKT-IKILLEYADMIFTYIFILEMLLKWMAYGF |
| XP_006153638.1 Na_x_ III *Tupaia chinensis* | FKCFIGLVTLLSTGALAFEDIYI--DQRKT-IKILLEYADMIFTYIFILEMLLKWMAYGF |
| XP_005907969.1 Na_x_ III *Bos mutus* | FKCFIGLVTLLSTGALAFEDIYI--DQRKT-IKILLEYADMIFTYIFILEMLLKWMAYGF |
| XP_004857664.1 Na_x_ III *Heterocephalus glaber* | FKCFIGLVTLLSTGALAFEDIYI--DQRKT-IKILLEYADMIFTYIFILEMLLKWMAYGF |
| XP_005393341.1 Na_x_ III *Chinchilla lanigera* | FKCFIGLVTLLSTGALAFEDIYI--DQRKT-IKILLEYADMIFTYIFILEMLLKWMAYGF |
| XP_003405861.1 Na_x_ III *Loxodonta Africana* | FKCFIGLVTLLSTATLAFEDIYI--NQRKT-IKILLEYADMIFTYIFILEILLKWMAYGF |
| XP_006867096.1 Na_x_ III *Chrysochloris asiatica* | FKCFIGLVTLLSTGALAFEDIYI--NQRKT-VKILLEYADMIFTYIFILEMLLKWMAYGF |
| XP_007945562.1 Na_x_ III *Orycteropus afer afer* | FKCFIGLVTLLSTGALAFEDIYI--NQRKT-IKILLEYADMIFTYIFILEMLLKWMAYGF |
| XP_007539379.1 Na_x_ III *Erinaceus europaeus* | FKCFIGIITLISTAALAFEDIYI--DQRKT-IKILLEYADMIFAYIFILEMLLKLVAYGF |
| XP_004674928.1 Na_x_ III *Condylura cristata* | FKCFIGLVTLVSAGALAFEDIYI--DQRKA-IKILLEYADMIFTYIFILEMLLKWMAYGF |
| XP_007074683.1 Na_x_ III *Panthera tigris altaica* | FKCFIGLVTLLSTGALAFEDIYI--DQRKT-IKILLEYADMIFTYIFILEMLLKWMAYGF |
| XP_006180754.1 Na_x_ III *Camelus ferus* | FKGFIGLVTLLSTGALAFEDIYI--DQRKT-IKILLEYADMIFTYIFILEMLLKWMAYGL |
| XP_007183279.1 Na_x_ III *B. acu. Scammoni* | FKCFIGLVTLLSTGALAFEDIYI--DQRKT-IKILLEYADMIFTYIFILEMLLKWMAYGF |
| XP_001496911.1 Na_x_ III *Equus caballus* | FQCFIGFVTLLSTGALAFEDIYI--DQRKT-IRILLEYADMIFTYVFVLEMLLKWMAYGL |
| XP_004428289.1 Na_x_ III *C. simum simum* | FKCFIGFVTLLSTGALAFEDIYI--DQRKT-IKILLEYADMIFTYIFILEMLLKWMAYGF |
| XP_002923846.1 Na_x_ III *Ailuropoda melanoleuca* | FKCFIGLVTLLSTGALAFEDIYI--DQRRT-IKILLEYADMIFTYIFILEMLLNWMAYGF |
| XP_006736092.1 Na_x_ III *Leptonychotes weddellii* | FKCFIGLVTLLSTGALAFEDIYI--DQRKT-IKILLEYADMIFTYIFILEMLLKWMAYGF |
| XP_008256919.1 Na_x_ III *Oryctolagus cuniculus* | FKCFIGLVTLFSTSALAFEDIYI--DQRKT-IKILLEYADMIFTYIFILEMLLKWIAYGF |
| XP_008582173.1 Na_x_ III *Galeopterus variegatus* | FKCFIGLVTLLSTGALAFEDIYI--DQKKT-IKILLEYADMIFTYIFILEMLLKWMAYGF |
| XP_004577381.1 Na_x_ III *Ochotona princeps* | FKCFIGLVTLFSTGTLAFEDIYI--DQKKT-IKILLEYADMTFTYIFVLEMLLKWMAYGF |
| AEW90239.1 Na_x_ III *Clytia hemisphaerica* | FDSFILLLILSSSFVLVFEDIRL--PERPQ-LQTALDILNIVFSVLFLIECILKIIGFGV |
| XP_028519297.1 Na_v_1.4 III *Aiptasia pallida* | FEWTILAIIMASSIALTFEDINL--PSRPK-LKEYLQYLNIFFAVTFSIEFLLKVLGLGV |
| AAA75572.1 Na_v_ III *Cyanea capillata* | FEFMILFLIAFSSLTLVFEDIHL--SKKVV-LKAFLDYCNYFFAIVFTLEFIIKLIGFGV |
| AAC38974.1 Na_v_ III *Polyorchis penicillatus* | FEGVILFLIAFSSLTLVFEDIDL--PKRPT-LEKFLQYCNYFFAVIFTVELLIKLFALGF |
| AEW90238.1 Na_v_2.5 III *Hydra vulgaris* | FEAVVLFLICVSSITLVFEDVYL--PSRPT-LKTFLQYCNYFFAFIFTLEFLIKLFALGF |
| XP_010200872.1 Na_v_1.5 III *Colius striatus* | FENFIIFIIILSSAALAFEDIHL--QERKT-VKIILERADKIFTYIFFMEMLLKWVAYGL |
| XP_008161265.1 Na_v_1.5 III *Chrysemys picta bellii* | FETFIIFIILLSSATLAFEDIYL--KEYAT-IQNLLKLADIIFAYIFLLEMFLKWVAYGF |
| AEX00073.1 Na_v_2.4 III *Nematostella vectensis* | FEGIILFLIAASSISLAFEDVYL--DSKPT-LKQVLQILNILFAVIFTVEMLLKWIGLGF |
| AEX00066.1 Na_v_2.2 III *Trichoplax adhaerens* | FEGIILLLIFASSVILALEDAYI--LQRQD-LARTIEALNVFFAIAFTVEMVLKWIGLGF |
| XP_002125324.3 Na_v_1.1 III *Ciona intestinalis* | FEWFILFLILSSTVCLAMEDIHL--NSDPV-RKLVLERLEYVFTALFTVEMVLKWLGIGP |
| BAA03398.1 Na_v_ III *Heterololigo bleekeri* | FETGVLVIIFASSILLAFEDIYL--NEKPR-LKLAIFYLDITFCLLFFLEMVLKLVALGF |
| ELT91324.1 Na_v_ III *Capitella teleta* | FEYLILVFIFASSVTLVFEDIYI--DQMPK-RKEILYYLDIVFVTIFVVEMFIKWIGLGF |
| ESO94539.1 Na_v_ III *Lottia gigantean* | FEYGILFVIFASSVTLAFEDVHL--EENQE-LKLALYYLNIIFCVIFVAEMLFKWFAYGL |
| EKC21550.1 Na_v_1.5 III *Crassostrea gigas* | FEYFILVCIGLSSMSLAFEDVYL--YTRPE-LEAALYYTNIIFAVLFTVEMLMKWVALGF |
| EEN42493.1 Na_v_ III *Branchiostoma floridae* | FETFIVGMILLSSLALAFEDIYL--KDRPA-LQLGLNIADRLFAVIFCCEMLIKWIAFGY |
| ESO08974.1 Na_v_ III *Helobdella robusta* | FETFIILMILASSLALAIEDKNI--DTKPL-LKHVLKVMDSVFTVIFVFEMIVKMAAYGL |
| XP_006818874.1 Na_v_ III *Saccoglossus kowalevskii* | FEGIVLFLIAASSISLIFEDIYL--NERAAIYRELLSYADIFFAIAFTIEMLLKWVGFGF |
| XP_793384.3 Na_v_ III *S. purpuratus* | FETFIIVVIFGSSFTLIFEDIYL--DLHPR-RQEILKILNYVFFGIFVVEMLIKWSGYGF |
| EFX89321.1 Na_v_ III *Daphnia pulex* | FEWSILILIFGSSITLCFEDIYL--EENPY-LMSILRWTNMAFAILFALEMIIKWFALGL |
| EHJ64356.1 Na_v_1.1 III *Danaus plexippus* | FEWFVLVLIFASSITLCFEDIHL--EKNIP-LKKILYWTNLSFCMIFVIEMFFKWIALGF |
| AFC61133.1 Na_v_1.1 III *Bombyx mori* | FEWFVLVLIFASSITLCFEDIHL--EKNKP-LKKILYWTNLGFCMIFIIEMFLKWIALGF |
| KFB48601.1 Na_v_ III *Anopheles sinensis* | FEWFVLVLIFASSITLCFEDIHL--DKNKE-LKRILYWTNLVFCLIFIIEMFLKWIALGF |
| EAA04706.4 Na_v_ III *Anopheles gambiae* | FEWFVLVLIFASSITLCFEDIHL--DKNKE-LKRILYWTNLVFCMIFIIEMFLKWIALGF |
| EDV37233.1 Na_v_ III *Drosophila ananassae* | FEWFVLVLIFASSITLCFEDINL--DKNKT-LKRVLYWINFSFCLIFVVEMILKWLALGF |
| AFH08262.1 Na_v_ III *Drosophila melanogaster* | FEWFVLVLIFASSITLCFEDINL--DKNKT-LKRVLYWINFSFCLIFVVEMILKWLALGF |
| EDW72405.1 Na_v_ III *Drosophila willistoni* | FEWFVLVLIFASSITLCFEDIYL--DSNKT-LKRVLYWTNFSFCLIFVVEMILKWLALGF |
| XP_005178206.1 Na_v_ III *Musca domestica* | ---FVLVLIFASSITLCFEDIYL--DSNKS-LKRILYWTNFSFCLIFVIEMVLKWLALGF |
| XP_004535967.1 Na_v_ III *Ceratitis capitata* | FEWFVLVLIFASSITLCFEDIYL--DSNKT-LKRILYWTNFSFCLIFVIEMILKWLALGF |
| XP_008196059.1 Na_v_ III *Tribolium castaneum* | FEWFILVLIFASSVTLCFEDIHL--DESPD-LKSVLYWTNLAFSIIFIIEMLLKWIALGF |
| AAK01090.1 Na_v_1.1 III *Blattella germanica* | FEWFILVLIFASSITLCFEDIYL--DQNLV-LKNVLYWTNLGFCALFSIEMMLKWLALGF |
| AGL91670.1 Na_v_ III *L. bostrychophila* | FEWVILVLIFSSSITLCFEDIYL--DQNKM-LKNILYWTNFVFCALFSIEMLLKWVALGF |
| EEB12429.1 Na_v_1.2 III *P. humanus corporis* | FEWIILVLIFSSSITLCFEDIYL--DNNKV-LKNILYWTNLGFCALFSIEMLLKWVALGF |
| AFC61134.1 Na_v_1.1 III *Nilaparvata lugens* | FEWFILVLIFSSSITLCFEDIYL--DENLF-LKNILYWTNFGFCAVFSVEMLLKWIALGF |
| XP_008545189.1 Na_v_ III *Microplitis demolitor* | FEWMILAFIFASSVTLCFEDIYL--DDNQF-LKRILYWTNLVFCLLFSIEMLLKWLALGF |
| XP_003704512.1 Na_v_ III *Megachile rotundata* | FEWMILVLIFASSITLCFEDIYL--DDNPF-LKKILYWTNLGFCALFSVEMLLKWLALGF |
| EZA62325.1 Na_v_ III *Cerapachys biroi* | FEWMILVLIFASSITLCFEDIYL--DDNPF-LKKILYWTNLGFCALFSVEMLLKWLALGF |
| XP_003697620.1 Na_v_ III *Apis florea* | FEWMILVLIFASSITLCFEDIYL--DDNPF-LKKILYWTNLGFCALFSIEMLLKWLALGF |
| XP_003493336.1 Na_v_ III *Bombus impatiens* | FEWMILVFIFASSITLCFEDIYL--DDNPF-LKKILYWTNLGFCALFSIEMLLKWLALGF |
| CBY22707.1 Na_v_ III *Oikopleura dioica* | FEMFIVLMIMLSSIALAFEDVNL--KDKPH-LQRGLKYSDKIFTYVFICEMILKWMGYGF |
| XP_007059504.1 Na_v_1.5 III *Chelonia mydas* | FESFIIFMILLSSGALAFEDIHL--QKREN-IKTMLEFLDKVFTYIFVFEMFLKWLAYGF |
| XP_003206989.1 Na_v_1.5 III *Meleagris gallopavo* | FESFIIFMILLSSGALAFEDIHI--NERQT-IKAVLLFLDRLFTFVFFLEMILKWVAYGF |
| XP_009084518.1 Na_v_1.5 III *Serinus canaria* | FESFIVFMILLSSGALAFEDIHI--HKRER-IQAILGFLDKMFTFIFILEMLLKWVAYGF |
| XP_008920014.1 Na_v_1.5 III *Manacus vitellinus* | FESFIIFMILLSSGALAFEDIHL--PKRVR-IQMILAFLDKMFTYIFVLEMLLKWVAYGF |
| XP_005512551.1 Na_v_1.5 III *Columba livia* | FESFIIFMILLSSGALAFEDIHI--NERKR-IKMILVFLDKLFTFIFVLEMLLKWVAYGF |
| XP_009479426.1 Na_v_1.5 III *Pelecanus crispus* | FESFIIFMILLSSGALAFEDIHI--NERQN-IKLMLMFLDKMFTFIFVLEMLLKWVAYGF |
| XP_010158440.1 Na_v_1.5 III *Eurypyga helias* | FETFIIFMILLSSGALAFEDIHI--NKRKS-IKMMLMFLDKLFTFIFVLEMLLKWVAYRF |
| XP_009286087.1 Na_v_1.5 III *Aptenodytes forsteri* | FESFIIFMILLSSGALVFEDIHI--NERRN-IKIMLAFLDKMFTFVFVLEMLLKWVAYGF |
| XP_009941400.1 Na_v_1.5 III *Opisthocomus hoazin* | FESFIIFMILLSSGALAFEDIHI--NERKS-IKAMLAFLDKLFTFIFVLEMLLKWVAYGF |
| XP_009465855.1 Na_v_1.5 III *Nipponia nippon* | FESFIIFMILLSSGALAFEDIHI--HERQS-IKIMLAFLDKMFTFIFVLEMLLKWVAYGF |
| XP_009962499.1 Na_v_1.5 III *Tyto alba* | FESFIIFMILLSSGALAFEDIHI--NERKS-IKIMLSFLDKMFTFIFVLEMLLKWVAYGF |
| XP_009642396.1 Na_v_1.5 III *Egretta garzetta* | FESFIIFMILLSSGALAFEDIHI--NERKS-IKIMLSFLDKMFTFIFVLEMLLKWVAYGF |
| XP_009068651.1 Na_v_1.5 III *Acanthisitta chloris* | FEYFVIFMIILSSAALAFEDIHL--QKRKK-VKIILEYADKIFTYVFFMEMLLKWVAYGL |
| DAA34928.1 Na_v_1.9 III *Gallus gallus* | FENFIIFVIVLSSAALAFEDIHL--KDRKT-VKKLLEYADMVFTYIFIMEMLLKWVAYGL |
| XP_007565048.1 Na_v_1.4 III *Poecilia formosa* | FKSFIIIMILLSSAVLAFEDIYI--EQRRD-IKIILDYADQVFNFVFVVEIIFKLVAYGC |
| ABA54922.1 Na_v_1.5 III *Danio rerio* | FETFIILMILLSSGALAFEDIYI--EQRKV-VKVVLEYADKVFSYIFVLEMFLKWIAYGF |
| XP_005475994.1 Na_v_1.4 III *Oreochromis niloticus* | FETFIIFMILLSSGALAFEDIYI--EKRKV-IKVVLEYADKVFSYIFVLEMFLKWIAYGF |
| XP_005813731.1 Na_v_1.4 III *Xiphophorus maculatus* | FETFIIFMILLSSGALAFEDIYI--ERRKV-IKVMLEYADKVFSYIFVLEMFLKWIAYGF |
| XP_007233940.1 Na_v_1.2 III *Astyanax mexicanus* | FETFIIFMILLSSGALAFEDIYI--EQRKV-VKIVLEYADKIFTYIFILEMTLKWIAYGF |
| XP_005946612.1 Na_v_1.5 III *Haplochromis burtoni* | FETFIIFMILLSSGALAFEDIYI--EQRRV-IKVVLEYADKIFTYIFILEMMLKWLAYGF |
| XP_009553849.1 Na_v_1.2 III *Cuculus canorus* | FESFIVFMILLSSATLAFEDIYI--EQRKT-VKIILDYADKIFTYVFILEMVLKWVAYGF |
| XP_005519819.1 Na_v_1.2 III *Pseudopodoces humilis* | FESFIVFMILLSSGALAFEDIYI--EKHKT-VKILLDYADKIFTYVFILEMLLKWVAYGF |
| XP_009086199.1 Na_v_1.2 III *Serinus canaria* | FESFIVFMILLSSGALAFEDIYI--EQRKT-IKVLLDYADKIFTYVFILEMVLKWVAYGF |
| XP_009994594.1 Na_v_1.2 III *Chaetura pelagica* | FETFIVFMILLSSAALAFEDIYI--EQRKT-IKVILEYADKIFTYVFILEMVLKWVAYGF |
| XP_009892890.1 Na_v_1.2 III *Charadrius vociferus* | FETFIVFMILLSSGALAFEDIYN--EQRKT-IRIILDYADKIFTYVFILEMVLKWVAYGF |
| XP_009639044.1 Na_v_1.2 III *Egretta garzetta* | FETFIVFMILLSSGALAFEDIYI--EQRKT-IKIILEYADKIFTYIFILEMVLKWVAYGF |
| XP_010121454.1 Na_v_1.2 III *Chlamydotis macqueenii* | FETFIVFMILLSSGALAFEDIYI--EQRKT-IKIILEYADKVFTYIFILEMVLKWVAYGF |
| XP_006180752.1 Na_v_1.7 III *Camelus ferus* | FESFIVLMILLSSGALAFEDIYI--EKKRT-IKTILEYADKIFTYIFILEMLLKWVAYGY |
| XP_004674637.1 Na_v_1.7 III *Condylura cristata* | FESFIVLMILLSSGALAFEDIYI--EKKKT-IKIVLDYADKIFTYVFILEMLLKWIAYGY |
| XP_007945734.1 Na_v_1.7 III *Orycteropus afer afer* | FESFIVLMILLSSGALAFEDIYI--EKKKT-IKIILEYADKIFTYVFILEMLLKWVAYGY |
| XP_006879389.1 Na_v_1.7 III *Elephantulus edwardii* | FESFIVLMILLSSGALAFEDIYI--EKKKT-IKIILEYADKIFTYVFILEMLLKWVAYGY |
| XP_003795197.1 Na_v_1.7 III *Otolemur garnettii* | FESFIVLMILLSSGALAFEDIYI--ERKKT-IKIILEYADKIFTYIFILELLLKWVAYGY |
| XP_006921216.1 Na_v_1.7 III *Pteropus alecto* | FESFIVLMILLSSGALAFEDIDI--EKKKT-IKIILEYADKIFTYIFILEMLLKWVAYGY |
| XP_009441903.1 Na_v_1.7 III *Pan troglodytes* | FESFIVLMILLSSGALAFEDIYI--ERKKT-IKIILEYADKIFTYIFILEMLLKWIAYGY |
| XP_004577152.1 Na_v_1.7 III *Ochotona princeps* | FESFIVLMILLSSGALAFEDIYI--EKKKT-IKIILEYADKIFTYIFILEMLLKWVAYGY |
| XP_008829101.1 Na_v_1.7 III *Nannospalax galili* | FESFIVLMILLSSGALAFEDIYI--EKKKT-IKIILEYADKIFTYIFILEMVLKWVAYGY |
| ELR47486.1 Na_v_1.7 III *Bos mutus* | FESFIVLMILLSSGALAFEDIYI--EKKKN-IKIILEYADKIFTYIFILEMLLKWVAYGY |
| AAA89159.1 Na_v_1 III *Oryctolagus cuniculus* | FESFIVLMILLSSGALAFEDIYI--EKKKT-IKIILEYADKIFTYIFILEMLLKWVAYGY |
| XP_004322655.1 Na_v_1.7 III *Tursiops truncatus* | FESFIVLMILLSSGALAFEDIYI--EKKKT-IKIILEYADKIFTYIFILEMLLKWIAYGY |
| ABB29444.1 Na_v_1.4 III *Tetraodon nigroviridis* | FESFIVFMILLSSGALAFEDIYL--EKHQL-IKTILEYADKVFTYVFVVEMVLKWFAYGF |
| ABB29442.1 Na_v_1.4 III *Takifugu rubripes* | FESFIVFMILLSSGALAFEDIYL--EKHQL-IKSILEYADKVFTYVFVMEMVLKWFAYGF |
| XP_007241580.1 Na_v_1.4 III *Astyanax mexicanus* | FETFIIFMILLSSGALAFEDIYL--EQRRT-IKIILEYADQVFTYIFVVEMLLKWCAYGF |
| XP_006003324.1 Na_v_1.4 III *Latimeria chalumnae* | FETFIIFMILLSSGALAFEDVYI--ERRRV-VKIILEYADKVFTYVFVVEMLLKWTAYGF |
| XP_002933087.2 Na_v_1.4 III *Xenopus tropicalis* | FETFIIFMILLSSGALAFEDIYI--EQRQV-IKTILEYADKVFTYIFVFEMLLKWTAYGF |
| XP_007628932.1 Na_v_1.4 III *Cricetulus griseus* | FETFIVFMILLSSGALAFEDIYI--EQRRV-IRTILEYADKVFTYIFILEMLLKWVAYGF |
| AAW68223.1 Na_v_1.4 III *Thamnophis sirtalis* | FETFIIFMILLSSGALAFEDIYI--ERRHT-IRTILEYADKIFTYVFILEMLLKWVAYGF |
| XP_009683652.1 Na_v_1.4 III *S. camelus australis* | FETFIVFMILLSSGALAFEDIHI--EQRRA-IRTILEYADKVFSYIFVIEMLLKWVAYGF |
| XP_010226082.1 Na_v_1.4 III *Tinamus guttatus* | FETFIVFMILLSSGALAFEDIHI--EQRKV-IRTILEYADKVFSYIFVIEMLLKWVAYGF |
| XP_007424896.1 Na_v_1.4 III *Python bivittatus* | FETFIIFMILLSSGALAFEDIYI--ERRHT-IRTILEFADKVFTYVFVIEMLLKWVAYGF |
| XP_008111415.1 Na_v_1.4 III *Anolis carolinensis* | FETFIIFMILLSSGALAFEDIYI--ERRPV-IRTILEYADKVFSYVFVIEMLLKWVAYGF |
| XP_006274845.1 Na_v_1.4 III *A. mississippiensis* | FETFIIFMILLSSGALAFEDIYI--EQRKV-IRTILEYADKIFSYVFVIEMLLKWVAYGF |
| XP_005283172.1 Na_v_1.4 III *Chrysemys picta bellii* | FETFIIFMILLSSGALAFEDIYI--EQRKV-IRTILEYADKVFSFVFVLEMLLKWVAYGF |
| XP_003768502.1 Na_v_1.4 III *Sarcophilus harrisii* | FETFIVFMILLSSGALAFEDIYI--EQRKV-IRTILEYADKVFSYVFVLEMLLKWVAYGF |
| XP_008931935.1 Na_v_1.4 III *Manacus vitellinus* | -----IFMILLSSGALAFEDIYI--EQRKV-IRTILEYADKVFSYIFVIEMLLKWVAYGF |
| DAA34926.1 Na_v_1.4 III *Gallus gallus* | FETFIVFMILLSSGALAFEDIYI--EQRKV-IRTILEYADKVFSYIFVIEMLLKWVAYGF |
| KFQ96635.1 Na_v_1.2 III *Nipponia nippon* | FETFIVFMILLSSGALAFEDIYI--EQRKV-IRTVLEYADKVFSYVFVIEMLLKWVAYGF |
| KFP89920.1 Na_v_1.2 III *Apaloderma vittatum* | FETFIVFMILLSSGALAFEDIYI--EKRKV-IRTILEYADKVFSYIFVIEMLLKWVAYGF |
| XP_005021806.1 Na_v_1.4 III *Anas platyrhynchos* | FETFIVFMILLSSGALAFEDIYI--EQRRV-IRTVLEYADKVFSYIFVIEMLLKWVAYGF |
| KFO60281.1 Na_v_1.2 III *Corvus brachyrhynchos* | FETFIVFMILLSSGALAFEDIYI--EQHKV-IRTILEYADKVFSYVFVIEMLLKWVAYGF |
| KFP55758.1 Na_v_1.2 III *Cathartes aura* | FETFIVFMILLSSGALAFEDIYI--EQRRV-IRTILEYADKVFSYVFVIEMLLKWVAYGF |
| XP_009565587.1 Na_v_1.4 III *Cuculus canorus* | FETFIVFMILLSSGALAFEDIYI--EQRKV-IRTILEYADKVFSYIFVIEMLLKWVAYGF |
| XP_009997626.1 Na_v_1.4 III *Chaetura pelagica* | FETFIVFMILLSSGALAFEDIYI--EQRKV-IRTILEYADKVFSYIFVIEMLLKWVAYGF |
| XP_009073953.1 Na_v_1.4 III *Acanthisitta chloris* | FETFIVFMILLSSGALAFEDIYI--EQRKV-IRTILEYADKVFSYIFVIEMLLKWVAYGF |
| XP_010131382.1 Na_v_1.4 III *B. rhinoceros silvestris* | FETFIVFMILLSSGALAFEDIYI--EQRKV-IRTILEYADKVFSYIFVIEMLLKWVAYGF |
| XP_009700369.1 Na_v_1.4 III *Cariama cristata* | FETFIVFMILLSSGALAFEDIYI--EQRKV-IRTILEYADKVFSYIFVIEMLLKWVAYGF |
| KFQ13298.1 Na_v_1.5 III *Leptosomus discolor* | FETFIVFMILLSSGALAFEDIYI--EQRKV-IRTILEYADKVFSYIFVIEMLLKWVAYGF |
| XP_010018959.1 Na_v_1.4 III *Nestor notabilis* | FETFIVFMILLSSGALAFEDIYI--EQRKV-IRTILEYADKVFSYIFVIEMLLKWVAYGF |
| XP_005508265.1 Na_v_1.4 III *Columba livia* | FETFIVFMILLSSGALAFEDIYI--EQRKV-IRTILEYADKVFSYIFVIEMLLKWVAYGF |
| XP_002939316.2 Na_v_1.2 III *Xenopus tropicalis* | FESFIIFMILLSSGALAFEDIYV--EQRRN-VKAILEYADKVFAYIFIMEMLLKWVAYGF |
| XP_004081947.1 Na_v_1.2 III *Oryzias latipes* | FESFIIFMILLSSGALAFEDVYS--EQRKT-IKIILEFADKMFTYIFILEMLLKWLAYGF |
| XP_008136988.1 Na_v_1.1 III *Eptesicus fuscus* | FETFIVFMILLSSGALAFEDIYI--DQRKT-IKTMLEYADKVFTYIFILEMLLKWVAYGY |
| XP_003795205.1 Na_v_1.1 III *Otolemur garnettii* | FETFIVFMILLSSGALAFEDIYI--DQRKT-IKTMLEYADKVFTYIFILEMLLKWVAYGY |
| XP_005986260.1 Na_v_1.6 III *Latimeria chalumnae* | FETFIIFMILLSSGALAFEDIYI--EQRRT-IRTMLEYADKVFTYIFIVEMLLKWIAYGY |
| DAA34919.1 Na_v_1.6 III *Xenopus tropicalis* | FETFIIFMILLSSGALAFEDVYI--EQRKT-IRTILEYADKVFTYIFILEMLLKWLAYGF |
| XP_005430143.1 Na_v_1.6 III *Geospiza fortis* | FETFIIFMILLSSGALAFEDIYI--EQRKT-IRTILEYADKVFTYIFILEMLLKWCAYGF |
| XP_006636602.1 Na_v_1.2 III *Lepisosteus oculatus* | FETFIIFMILLSSGALAFEDIYI--EQRKT-IKTMLEYADKVFTYIFILEMLLKWVAYGF |
| XP_006003661.1 Na_v_1.2 III *Latimeria chalumnae* | FETFIIFMILLSSGALAFEDIYI--EQRRT-IKTMLEYADKVFTYIFILEMLLKWVAYGF |
| XP_008113359.1 Na_v_1.2 III *Anolis carolinensis* | FETFIVFMILLSSGALAFEDIYI--EQRKT-IKTMLEYADKVFTYIFILEMLLKWVAYGF |
| XP_006137390.1 Na_v_1.3 III *Pelodiscus sinensis* | FETFIVFMILLSSGALAFEDIYI--EQRKT-IKTMLEYADKVFTYIFILEMLLKWVAYGF |
| XP_009674477.1 Na_v_1.2 III *S. camelus australis* | FETFIVFMILLSSGALAFEDIYI--EQRKT-IKTMLEYADKVFTYIFILEMLLKWVAYGF |
| XP_001367154.1 Na_v_1.3 III *Monodelphis domestica* | FETFIVFMILLSSGALAFEDIYI--EQRKT-IKTMLEYADKVFTYIFILEMLLKWVAYGF |
| XP_008997034.1 Na_v_1.3 III *Callithrix jacchus* | FETFIVFMILLSSGALAFEDIYI--EQRKT-IKTMLEYADKVFTYIFILEMLLKWVAYGF |
| XP_006160314.1 Na_v_1.3 III *Tupaia chinensis* | FETFIVFMILLSSGALAFEDIYI--EQRKT-IKTMLEYADKVFTYIFILEMLLKWVAYGF |
| XP_004601166.1 Na_v_1.3 III *Sorex araneus* | FETFIVFMILLSSGALAFEDIYI--EQRKT-IKTMLEYADKVFTYIFILEMLLKWVAYGF |
| XP_007059504.1 Na_v_1.5 I *Chelonia mydas* | FTMFIMCTILTNCVFMAISETPG--KSKSPESPVWNKYVEYTFTGIYTFESLIKILARGF |
| XP_003206989.1 Na_v_1.5 I *Meleagris gallopavo* | FTWFIICTIITNCVFMARNESS-----RS-TSPSWNKYVEFTFTGIYTFESLIKILARGF |
| XP_009084518.1 Na_v_1.5 I *Serinus canaria* | FTWFIMCTIITNCVFMALTESS-----KSS-SPSWNTYVEFTFTGIYTFESLIKILATGF |
| XP_008920014.1 Na_v_1.5 I *Manacus vitellinus* | FTLFIMCTILTNCVFMALTESS-----KTTSPSKLLKYVEFTFTGIYTFESLIKILARGF |
| XP_009286087.1 Na_v_1.5 I *Aptenodytes forsteri* | FTYFIMCTIITNCAFMALTESS-----KASASPSWNKYVEFTFTGIYTFESLIKILARGF |
| XP_009642396.1 Na_v_1.5 I *Egretta garzetta* | FTSFIICTIITNCVFMALTESY-----KASPSSSWNKYVEFTFTGIYTFESLIKILARGF |
| XP_009479426.1 Na_v_1.5 I *Pelecanus crispus* | FTLFIMCTILTNCVFMALTESS-----KASPSPSWNKYVEFTFTGIYTFESLIKILARGF |
| XP_009465855.1 Na_v_1.5 I *Nipponia nippon* | FTLFIMCTIITNCVFMALTESF-----KASASPSWNKYVEFTFTGIYTFESLIKILARGF |
| XP_010158440.1 Na_v_1.5 I *Eurypyga helias* | FTWFIMCTIITNCAFMALTESS-----KSSPSPLWNKYVEFTFTGIYTFESLIKILARGF |
| XP_009962499.1 Na_v_1.5 I *Tyto alba* | FTLFIICTIITNCVFMALTESS-----RTSPSPSWNKYVEFTFTGIYTFESLIKILARGF |
| XP_005512551.1 Na_v_1.5 I *Columba livia* | FTYFIMCTITTNCVFMALTESA-----KSSSSPSWNKYVEFTFTGIYTFESLIKILARGF |
| XP_009941400.1 Na_v_1.5 I *Opisthocomus hoazin* | FTWFIMCTIITNCVFMALTESS-----KTSPSPSWNKYVEFTFTGIYTFESLIKILARGF |
| ABB29442.1 Na_v_1.4 I *Takifugu rubripes* | FSLFIMATILTNCAFMTLSD-----------PPAWSKTMEYVFTFIYTFEATIKILSRGF |
| ABB29444.1 Na_v_1.4 I *Tetraodon nigroviridis* | FSLFIMATILTNCVFMTLSD-----------PPAWSKTVEYVFTFIYTFEATIKVVSRGF |
| AAW68223.1 Na_v_1.4 I *Thamnophis sirtalis* | FSMFIMITILANCVFMTMSN-----------PPIWAKDVEYTFTGIYTFEAMIKVLTRGF |
| XP_008111415.1 Na_v_1.4 I *Anolis carolinensis* | FSMFIMITILTNCVFMAMSN-----------PPSWAKNVEYTFTGIYTFESTIKILARGF |
| XP_006003324.1 Na_v_1.4 I *Latimeria chalumnae* | FSFFIMITILTNCVFMTMSA-----------PPPWAKNVEYTFTGIYTFESMIKILARGF |
| XP_007424896.1 Na_v_1.4 I *Python bivittatus* | FSMFIMITILANCFFMTLSD-----------PPPWAKNVEYTFTGIYTFEAMIKVLARGF |
| XP_002933087.2 Na_v_1.4 I *Xenopus tropicalis* | FSLFIMFTILTNCVFMTMSD-----------PPPWSKNVEYTFTGIYTFESLIKILARGF |
| XP_006274845.1 Na_v_1.4 I *A. mississippiensis* | FSMFIMITILTNCVFMTMSD-----------PPPWSKNVEYTFTGIYTFESMIKILARGF |
| XP_005283172.1 Na_v_1.4 I *Chrysemys picta bellii* | FSMFIMITILTNCVFMAMSD-----------PPPWAKNVEYTFTGIYTFESMIKIVARGF |
| XP_007241580.1 Na_v_1.4 I *Astyanax mexicanus* | FSMFIMVTILSNCVFMTMSN-----------PPEWSKIMEYVFTGIYTFEALIKVLSRGF |
| XP_003768502.1 Na_v_1.4 I *Sarcophilus harrisii* | FSLFIMITILANCVFMTMSE-----------PPPWSKNVEYTFTGIYTFESLIKMLSRGF |
| XP_007628932.1 Na_v_1.4 I *Cricetulus griseus* | FSMFIMITILTNCVFMTMSN-----------PPSWSKDVEYTFTGIYTFESLIKMLARGF |
| XP_008931935.1 Na_v_1.4 I *Manacus vitellinus* | FSMFIMITILTNCVFMTMNN-----------PPPWSKNVEYAFTGIYTFESLIKILSRGF |
| XP_010131382.1 Na_v_1.4 I *B. rhinoceros silvestris* | FGMFIMITILTNCVFMTLSN-----------PPTWSKNVEYTFTGIYTFESLIKVLSRGF |
| XP_010018959.1 Na_v_1.4 I *Nestor notabilis* | FSMFIMITILTNCVFMTLSN-----------PPAWSKNVEYTFTGIYTFESLIKVLSRGF |
| XP_009073953.1 Na_v_1.4 I *Acanthisitta chloris* | FSMFIMITILTNCVFMTMSN-----------PPAWSKNVEYAFTGIYTFESLIKILSRGF |
| XP_010226082.1 Na_v_1.4 I *Tinamus guttatus* | FSMFIMITILTNCVFMTMSN-----------PPAWSKNVEYAFTGIYTFESLIKILSRGF |
| XP_009683652.1 Na_v_1.4 I *S. camelus australis* | FSMFIMITILTNCVFMTMSN-----------PPAWSKNVEYTFTGIYTFESLIKILSRGF |
| XP_005021806.1 Na_v_1.4 I *Anas platyrhynchos* | FSMFIMITILTNCVFMTLSN-----------PPAWSKNVEYTFTGIYTFESLIKILSRGF |
| XP_009565587.1 Na_v_1.4 I *Cuculus canorus* | FSMFIMITILTNCVFMTLSN-----------PPAWSKNVEYTFTGIYTFESLIKILSRGF |
| XP_009700369.1 Na_v_1.4 I *Cariama cristata* | FSMFIMITILTNCVFMTLSN-----------PPAWSKNVEYTFTGIYTFESLIKILSRGF |
| XP_005508265.1 Na_v_1.4 I *Columba livia* | FSMFIMITILTNCVFMTMSN-----------PPAWSKNVEYTFTAIYTFESLIKILSRGF |
| DAA34926.1 Na_v_1.4 I *Gallus gallus* | FSMFIMITILTNCVFMTLSN-----------PPAWSKNVEYTFTGIYTFESLIKILSRGF |
| KFQ13298.1 Na_v_1.5 I *Leptosomus discolor* | FSMFIMITILTNCVFMTMSN-----------PPAWSKNVEYTFTGIYTFESLIKILSRGF |
| XP_009997626.1 Na_v_1.4 I *Chaetura pelagica* | FSMFIMITILTNCVFMTLSN-----------PPAWSKNVEYTFTGIYTFESLIKILSRGF |
| KFO60281.1 Na_v_1.2 I *Corvus brachyrhynchos* | FSMFIMITILTNCVFMTLSN-----------PPAWSKNVEYTFTGIYTFESLIKILSRGF |
| KFP55758.1 Na_v_1.2 I *Cathartes aura* | ALMFIMITILTNCVFMTMSN-----------PPAWSKNVEYTFTGIYTFESLIKILSRGF |
| KFQ96635.1 Na_v_1.2 I *Nipponia nippon* | SLMFIMITILTNCVFMTLSN-----------PPAWSKNVEYTFTGIYTFESLIKILSRGF |
| KFP89920.1 Na_v_1.2 I *Apaloderma vittatum* | PLMFIMITILTNCVFMTMSN-----------PPAWSKNVEYTFTGIYTFESLIKVLSRGF |
| XP_004081947.1 Na_v_1.2 I *Oryzias latipes* | FNVLIMCTILTNCALMTRSSSA-------DSNSTWTKPVEYTFTAIYTFESLVKILARGF |
| XP_005475994.1 Na_v_1.4 I *Oreochromis niloticus* | FSLFIMFTILTNCFFMAMSD-----------PPTWTKYLEYTFTGIYTFESAIKILARGF |
| XP_005813731.1 Na_v_1.4 I *Xiphophorus maculatus* | FSYFIMFTILTNCFFMAMSD-----------PPTWTKYLEYTFTGIYTFESAIKIFARGF |
| XP_005946612.1 Na_v_1.5 I *Haplochromis burtoni* | FSLFIMCTILTNCCFMAMSE-----------PEYWAKYLEYTFTGIYTFESLIKILARGF |
| ABA54922.1 Na_v_1.5 I *Danio rerio* | FSLFIMCTILTNCCFMAMSD-----------PPLWTKYLEYTFTGIYTFESLIKILARGF |
| XP_007233940.1 Na_v_1.2 I *Astyanax mexicanus* | FSLFIMCTILTNCCFMAMSE-----------PAPWAKYVEYTFTGIYTFESLIKILARGF |
| XP_009639044.1 Na_v_1.2 I *Egretta garzetta* | FSMLIMLTILTNCVFMTWNN-----------LPDWTKNVEYTFTGIYTFEFLVKILARGF |
| XP_010121454.1 Na_v_1.2 I *Chlamydotis macqueenii* | FSMLIMLTILTNCVFMTWKN-----------LPDWTKNVEYTFTGIYTFEFLVKILARGF |
| XP_009994594.1 Na_v_1.2 I *Chaetura pelagica* | FSILIMLTILTNCVFMTWTN-----------LPEWTKIVEYTFTGIYTFEFLVKILARGF |
| XP_009553849.1 Na_v_1.2 I *Cuculus canorus* | FSIFIMLTILTNCVFMTWSN-----------LPDWTKNVEYTFTGIYTFEFLVKILARGF |
| XP_009892890.1 Na_v_1.2 I *Charadrius vociferus* | FSILIMLTILTNCVFMTWTN-----------LPEWTKNVEHTFTGIYTFEFLVKILARGF |
| XP_005519819.1 Na_v_1.2 I *Pseudopodoces humilis* | FNKIIMLTILANCVLMTWRN-----------LPEWAKKVEYTFTGIYTFEFLVKIFARGF |
| XP_009086199.1 Na_v_1.2 I *Serinus canaria* | FNKVIMLTILANCVFMTWRN-----------LPEWAKNVEYTFTGIYTFEFLVKVFARGF |
| XP_008113359.1 Na_v_1.2 I *Anolis carolinensis* | FSMLIMCTILTNCVFMTMSN-----------PPEWTKNVEYTFTGIYTFESLIKILARGF |
| DAA34919.1 Na_v_1.6 I *Xenopus tropicalis* | FSMIIMCTILTNCVFMTFSN-----------PPEWSKQVEYTFTGIYTFESLVKIIARGF |
| XP_005986260.1 Na_v_1.6 I *Latimeria chalumnae* | FSMIIMCTILTNCVFMTFSN-----------PPEWSKQVEYTFTGIYTFESVVKIIARGF |
| XP_005430143.1 Na_v_1.6 I *Geospiza fortis* | FSMIIMCTILTNCVFMTFSN-----------PPEWSKNVEYTFTGIYTFESLVKIIARGF |
| XP_008997034.1 Na_v_1.3 I *Callithrix jacchus* | FSMLIMCTILTNCIFMTLYN-----------PPEWTKIVEYTFTGIYTFESLVKILARGF |
| XP_004674637.1 Na_v_1.7 I *Condylura cristata* | FSMLIMCTILTNCIFMTLNN-----------PPDWTKNVEYTFTGIYTFESLIKILARGF |
| XP_006180752.1 Na_v_1.7 I *Camelus ferus* | FSMLIMCTILTNCIFMTLSS-----------PPDWTKNVEYTFTGIYTFESLVKILARGF |
| XP_008829101.1 Na_v_1.7 I *Nannospalax galili* | FSMLIMCTILTNCIFMTLSS-----------PPDWTKNVEYTFTGIYTFESLIKILARGF |
| ELR47486.1 Na_v_1.7 I  *Bos mutus* | FSMLIMCTILTNCIFMTMSN-----------PPDWTKNVEYTFTGIYTFESLVKILARGF |
| XP_003795197.1 Na_v_1.7 I *Otolemur garnettii* | FSMLIMCTILTNCIFMTMSN-----------PPEWTKNVEYTFTGIYTFESLVKILARGF |
| AAA89159.1 Na_v_1 I *Oryctolagus cuniculus* | FSMLIMCTILTNCIFMTMNN-----------PAEWTKNVEYTFTGIYTFESLVKIFARGF |
| XP_006879389.1 Na_v_1.7 I *Elephantulus edwardii* | FSMLIMCTILTNCIFMTMSN-----------PPDWTKNVEYTFTGIYTFESLVKILARGF |
| XP_004577152.1 Na_v_1.7 I *Ochotona princeps* | FSMLIMCTILTNCIFMTMNN-----------PPDWTKNVEYTFTGIYTFESLVKILARGF |
| XP_009441903.1 Na_v_1.7 I *Pan troglodytes* | FSMLIMCTILTNCIFMTMNN-----------PPDWTKNVEYTFTGIYTFESLVKILARGF |
| XP_006921216.1 Na_v_1.7 I *Pteropus alecto* | FGMLIMCTILTNCIFMTMNN-----------PPDWTKNVEYTFTGIYTFESLIKILARGF |
| XP_007945734.1 Na_v_1.7 I *Orycteropus afer afer* | FNMLIMCTILTNCIFMTMSN-----------PPDWTKNVEYTFTGIYTFESLIKILARGF |
| XP_006003661.1 Na_v_1.2 I *Latimeria chalumnae* | FSMLIMCTILTNCVFMTMSN-----------PPEWAKNVEYTFTGIYTFESLIKILARGF |
| XP_006636602.1 Na_v_1.2 I *Lepisosteus oculatus* | FSMLIMCTILTNCSFMTLSN-----------PPDWAKNVEYTFTGIYTFESLIKILARGF |
| XP_002939316.2 Na_v_1.2 I *Xenopus tropicalis* | FNMLIMCTILTNCVFMTLSN-----------PPEWTKNVEYTFTGIYTLESLIKILARGF |
| XP_008136988.1 Na_v_1.1 I *Eptesicus fuscus* | FSMLIMCTILTNCVFMTMSN-----------PPEWTKNVEYTFTGIYTFESLIKIIARGF |
| XP_003795205.1 Na_v_1.1 I *Otolemur garnettii* | FSMLIMCTILTNCVFMTMSN-----------PPDWTKNVEYTFTGIYTFESLIKIIARGF |
| XP_004322655.1 Na_v_1.7 I *Tursiops truncates* | FSMLIMCTILTNCVFMTMSN-----------PPDWTKNVEYTFTGIYTFESLIKIIARGF |
| XP_006137390.1 Na_v_1.3 I *Pelodiscus sinensis* | FSMLIMCTILTNCVFMTMSN-----------PPDWTKNVEYTFTGIYTFESLIKILARGF |
| XP_006160314.1 Na_v_1.3 I *Tupaia chinensis* | FSMLIMCTILTNCVFMTLSN-----------PPDWTKNVEYTFTGIYTFESLIKILARGF |
| XP_004601166.1 Na_v_1.3 I *Sorex araneus* | FSMLIMCTILTNCVFMTLSN-----------PPDWTKNVEYTFTGIYTFESLIKILARGF |
| XP_009674477.1 Na_v_1.2 I *S. camelus australis* | FSMLIMCTILTNCVFMTMSN-----------PPDWTKNVEYTFTGIYTFESLIKILARGF |
| XP_001367154.1 Na_v_1.3 I *Monodelphis domestica* | FSMLIMCTILTNCVFMTMSN-----------PPEWTKNVEYTFTGIYTFESLIKILARGF |
| CBY22707.1 Na_v_ I *Oikopleura dioica* | FNNLIMLTILTNCGFMIKEDPPQ--W--------VNDYVERVFLAIYTLESTVKILSRGM |
| BAA03398.1 Na_v_ I *Heterololigo bleekeri* | FDYFLMFTIMINCVFLAMPDI----S----------EFAEYIFLGIYTMEMAIKLVAGGF |
| ESO94539.1 Na_v_ I *Lottia gigantean* | FDYFVILTIIVNCVFMTMPDLQI--T----------ETLEYIFLAIYVVEMLIKVTARGF |
| EKC21550.1 Na_v_1.5 I *Crassostrea gigas* | FDYLVILTILCNCVFLAMPDDPA--S----------ETAEYVFLGIYTMECVVKILARGL |
| XP_002125324.3 Na_v_1.1 I *Ciona intestinalis* | FDAIVMTTILVNCVFLGLNEE-----------I---AAAEYLFTALYTFEMVVKMVGRGF |
| ESO08974.1 Na_v_ I *Helobdella robusta* | PFLTVMVVILVNCVFMAIKEE-----------F---QAAEKTFTGIYTVEGSVKMLARGF |
| EEN42493.1 Na_v_ I *Branchiostoma floridae* | PFH-VMGATFINCVFMTMTAP-----------P---EMTEYVFTGVYSFEMIVKILARGF |
| AEX00066.1 Na_v_2.2 I *Trichoplax adhaerens* | FDFFVLLIILSNCVLLAFRPEEI--S--S------YNTIEIGYNVVYTIEMTLKIIARGF |
| AAA75572.1 Na_v_ I *Cyanea capillata* | FEIFILLTIITNCVFMALSDP-----------P---KESEYVFAAIYTFEVIVKISAKGF |
| AAC38974.1 Na_v_ I *Polyorchis penicillatus* | FEIGILLTIVTNCVFMALSDP-----------P---AEAEFIFAAIYTFEMVIKILAKGF |
| AEW90238.1 Na_v_2.5 I *Hydra vulgaris* | FEVFILLTIVTNCVFMALSHP-----------P---QLSEYVFAAIYTIEMFIKILAKGF |
| AEW90239.1 Na_x_ I *Clytia hemisphaerica* | FEFFVILTILVNCVFLALNDA-----------P---EEAEYFFTAIYTTEMVLKIIAKGF |
| AEX00073.1 Na_v_2.4 I *Nematostella vectensis* | FEMFVLLTILVNCVFLAMTNP-----------P---EQPEYVFAAIYTIEMFCKIIAKGF |
| XP_028519297.1 Na_v_1.4 I *Aiptasia pallida* | FEFFILLTIIVNCIFLALRDA-----------P---EQPEYVFAAIYTFEMLLKIIAKGL |
| XP_793384.3 Na_v_ I *S. purpuratus* | FDLFVILTIIANCVFLMLDTPRP--P--EVEEPTYLKIAEYIFTTIYSVEMFVKIFARGF |
| ELT91324.1 Na_v_ I *Capitella teleta* | FDYCVIATILINCVFLALPPQ---------------GWDTYIFTAIYTFECIVKLLARGF |
| XP_006818874.1 Na_v_ I *Saccoglossus kowalevskii* | FDIVVMLTILANCVFLGLNAE-----------NKYAVYAEYVFTGIYTFEMLVKILARGF |
| EFX89321.1 Na_v_ I *Daphnia pulex* | FDYVVMTTILLNCIFLAMTEP-----------V---EQAEYVFLGIYSCEMVIKAVAKGL |
| AFH08262.1 Na_v_ I *Drosophila melanogaster* | FDYCVMATILFNCIFLAMTET-----------V---EEAEYIFLAIYSIEMVIKIIAKGF |
| XP_005178206.1 Na_v_ I *Musca domestica* | -----MATILFNCIFLAMTET-----------V---EEAEFVFLAIYTIEMVIKIIAKGF |
| XP_004535967.1 Na_v_ I *Ceratitis capitata* | FDYCVMATILFNCIFLAMTET-----------V---EEAEYIFLAIYSIEMVIKIVAKGF |
| EDV37233.1 Na_v_ I *Drosophila ananassae* | FDYCVMATILFNCIFLAMTET-----------V---EEAEYIFLAIYSIEMVIKIIAKGF |
| EDW72405.1 Na_v_ I *Drosophila willistoni* | FDYCVMATILFNCIFLAMTET-----------V---EEAEYIFLAIYSIEMVIKIIAKGF |
| EAA04706.4 Na_v_ I *Anopheles gambiae* | FDYFVMATILFNCIFLAMSET-----------I---EEAEYIFLAIYTSEMIIKMIAKGF |
| AGL91670.1 Na_v_ I *L. bostrychophila* | FDYVVMATILLNCVFLAMTEP-----------I---EEAEFVFLAIYTAEMIIKSVAKGF |
| EEB12429.1 Na_v_1.2 I *P. humanus corporis* | FDYVVMTTILLNCVFLAMTEP-----------V---EEAEFVFLAIYTAEMIIKAIAKGF |
| AFC61133.1 Na_v_1.1 I *Bombyx mori* | FDYFVMATILLNCVFLAMSET-----------I---EEAEYIFLAIYTAEMIIKCIAKGF |
| XP_003704512.1 Na_v_ I *Megachile rotundata* | FDYMVMATIILNCAFLAMTET-----------I---EEAEYIFLAIYTAEMVIKSIAKGF |
| XP_003697620.1 Na_v_ I *Apis florea* | FDYVVMATIILNCAFLAMTET-----------I---EEAEYIFLAIYTAEMVIKSIAKGF |
| XP_003493336.1 Na_v_ I *Bombus impatiens* | FDYIVMATIILNCAFLAMTET-----------I---EEAEYIFLAIYTAEMVIKSIAKGF |
| KFB48601.1 Na_v_ I *Anopheles sinensis* | -----MATILFNCIFLAMSET-----------I---EEAEYIFLAIYTSEMIIKMIAKGF |
| XP_008545189.1 Na_v_ I *Microplitis demolitor* | ---MVMLTIVLNCVFLGMTEP-----------I---EEAEYIFLAIYTLEMVIKSIAKGF |
| AFC61134.1 Na_v_1.1 I *Nilaparvata lugens* | FDYVVMTTILLNCVFLAKTEP-----------L---EEAEYIFLAIYTAEMIIKSVAKGF |
| AAK01090.1 Na_v_1.1 I *Blattella germanica* | FDYVVMITILLNCVFLAMTDT-----------V---EEAEYIFLAIYTAEMIIKSIAKGF |
| XP_008196059.1 Na_v_ I *Tribolium castaneum* | -----MATILLNCVFLAMAET-----------I---EEAEYIFLAIYTAEMIIKSIAKGF |
| EZA62325.1 Na_v_ I *Cerapachys biroi* | FDYVVMATILLNCVFLAMTET-----------V---EEAEYIFLAIYTAEMVIKSIAKGF |
| EHJ64356.1 Na_v_1.1 I *Danaus plexippus* | -----MATILLNCVFLAMSET-----------I---EEAEYIFLAIYTAEMIIKCIAKGF |
| XP_007565048.1 Na_v_1.4 I *Poecilia formosa* | FSFFIIATILTDCVFLAMSDPP-----------EWSKTVEYVVMVIYTFEVIIKVLSRGF |
| XP_008161265.1 Na_v_1.5 I *Chrysemys picta bellii* | FTAFIIFTVLLNCVMMIPANFA-----------KININIEFTFAGIYTCEILIKVLASGF |
| XP_009068651.1 Na_v_1.5 I *Acanthisitta chloris* | FFAIITCTVLLNCAVMTQPQLL-----------ESLNWTEYVFTAIYSTEIGIKIMARGF |
| XP_010200872.1 Na_v_1.5 I *Colius striatus* | FVGFITCTVVLNCASMAVKDFP-----------EALSWTEYLFTGIYTVEILIKVVAKGF |
| DAA34928.1 Na_v_1.9 I *Gallus gallus* | FVGFITCTVILNCASMAMNNYP-----------LDFNGTEHIFTAIYTGEILIKVLARGL |
| XP_003766750.1 Na_v_1.7 I *Sarcophilus harrisii* | FNMILMGTFLFNCIFLTNLNPP-----------DWIFYFELSFIGLYTFEILIKILARGF |
| XP_007539379.1 Na_x_ I *Erinaceus europaeus* | FRMSMLIIVLTDCLFMSMSNMP-----------EWGAVLQTAFLGIYIFEIFLKMIARGI |
| XP_004674928.1 Na_x_ I *Condylura cristata* | FQLSILISVLIDCIFMPMTELP-----------EWGPALQTTLLGIYTFEILVKLIARGI |
| XP_008136835.1 Na_x_ I *Eptesicus fuscus* | FRLLILISVLIDCTFMSLTERP-----------KWGPELQITLLAIYTFEILVKLIARGI |
| XP_002923846.1 Na_x_ I *Ailuropoda melanoleuca* | FRLFILISVLIDCIFMSLTEMP-----------KWGPALQNTLLGIYTFEILIKLIARGI |
| XP_006736092.1 Na_x_ I *Leptonychotes weddellii* | FRLFILISVLTDCIFMSVTEMP-----------KWGPILQNTLLGIYTFEILIKLIARGI |
| XP_005907969.1 Na_x_ I *Bos mutus* | FRLFILVSVLTDGIYMSMTDLP-----------KWGPALQNTLLGIYTFEILVKLIARGI |
| XP_006180754.1 Na_x_ I *Camelus ferus* | FQLLILISVLVDCIFMSMTNVP-----------RWWPALQNTLLGIYTFEILIKLIARGI |
| XP_007074683.1 Na_x_ I *Panthera tigris altaica* | FRLFILISVLIDCMFMPMTELP-----------KWGPALQNTLLGIYTFEILIKLIARGI |
| XP_007183279.1 Na_x_ I *B. acu. Scammoni* | FRLLILISILIDCIFMSMTDLP-----------KWGPALQNTLLGIYTFEVLVKLIARGI |
| XP_001496911.1 Na_x_ I *Equus caballus* | FRLLILTSVLIDCVFMTMINLP-----------KWWPALQDTLLGIYTFEILVKLIARGI |
| XP_004428289.1 Na_x_ I *C. simum simum* | FRLLILISVLIDCIFMTMTDLP-----------KWWPALQNTLLGIYTFEILVKLIGRGI |
| XP_008256919.1 Na_x_ I *Oryctolagus cuniculus* | FQYLILICVLIDCTFLSMTNMP-----------KWGTAVENTLLGVYTFEIFVKLLARGI |
| XP_004857664.1 Na_x_ I *Heterocephalus glaber* | FQLLILISVLTDCILMTMNNLP-----------EWGPALEHTLLGIYTFEILVKVIARGI |
| XP_005393341.1 Na_x_ I *Chinchilla lanigera* | FQLSILIGVLTDCILMTMINLP-----------EWGPTAEHTLLGIYTFEILVKIIARGI |
| XP_004577381.1 Na_x_ I *Ochotona princeps* | FRYLILISVLTDCIFMSMSNLP-----------KWGPAIEHTLLGIYTFEIIVKLIARGL |
| XP_005315754.1 Na_x_ I *I. tridecemlineatus* | FRLLILISVLTDCIFMTMTNWS-----------IWITAIENTLLGIYTFEILVKIIARGF |
| XP_008582173.1 Na_x_ I *Galeopterus variegatus* | FRLLILISVLTDCMFMTMNNLP-----------KWGPAVENTLLGIYTFEILVKLFARGI |
| XP_006153638.1 Na_x_ I *Tupaia chinensis* | FRLFILISVLTDCILMSMINLP-----------QWGPALEHSLLGIYTFEILVKLLARGV |
| XP_010350732.1 Na_x_ I *S. bol. boliviensis* | FRLFILISVLTDCIFMSFTDLP-----------KWRPVVENTLLGIYTFEILVKLFARGV |
| XP_010363599.1 Na_x_ I *Rhinopithecus roxellana* | FRLFILISVLLDCIFMSLTNLP-----------KWIPVLENTLLGIYTFEILVKLFARGV |
| XP_002812597.1 Na_x_ I *Pongo abelii* | FRLFILISVLIDCIFMSLTNLP-----------KWRPVLENTLLGIYTFEILVKLFARGV |
| XP_006867096.1 Na_x_ I *Chrysochloris asiatica* | FRLLILISILTDCILMTMTTFS-----------YWRTILENTLLGIYTFEILVKLIARGI |
| XP_003405861.1 Na_x_ I *Loxodonta Africana* | FRMLILISVLTDCIFMTMTNFA-----------DWRALLENALLGIYTFEILVKLIARGI |
| XP_007945562.1 Na_x_ I *Orycteropus afer afer* | FRLLILISVLTDCILMTMTDFV-----------DWKAILENALLGIYTFEILVKLIARGI |
| XP_003766750.1 Na_v_1.7 IV *Sarcophilus harrisii* | FEITILVLICFNMIIMMVETDTE-----SVHKAGILYCINLVFIGLFAGECLLKLIGLRH |
| XP_007539379.1 Na_x_ IV *Erinaceus europaeus* | FNIIILVLICVQAIVMMTESDEQ-----STSMVLAQSWIDLILVILYTVECVLKLTAFRC |
| XP_003405861.1 Na_x_ IV *Loxodonta Africana* | FNITIMVLIFVHAITIMMKSDDE-----SPQMKTVFYWINTVFVVLYTVECLLKLISFRC |
| XP_006867096.1 Na_x_ IV *Chrysochloris asiatica* | FNIIIMVLICTHALSLMMESDDG-----SLQMQTAFFWINTIFVLIYAGECLLKLISFRC |
| XP_007945562.1 Na_x_ IV *Orycteropus afer afer* | FSIIIMVLIFAHALILMMESEDE-----SPQMEIALYWINTIFVLLYTGECLLKLISFRC |
| XP_006153638.1 Na_x_ IV *Tupaia chinensis* | FNAVIVVLTCLQALSIMIQYDEQ-----SPRMDTAVFWIDCIFVMLYTVECVLKLISFHC |
| XP_008582173.1 Na_x_ IV *Galeopterus variegatus* | FNLVIVVLICFQVITIMIQSDEQ-----SPQMETALYWVNSIFVILYTTECVMKLVSFRC |
| XP_008256919.1 Na_x_ IV *Oryctolagus cuniculus* | FNVVIIVLICSQVITFMIQSDEQ-----SPQVERAVYWISSVFVMLYTLECLLKLVSFLC |
| XP_010363599.1 Na_x_ IV *Rhinopithecus roxellana* | FNVIVMVLICFQAIAMMIDTEGQ-----SLEMYIALYWINSIFVMLYTMECILKLIALRC |
| XP_002812597.1 Na_x_ IV *Pongo abelii* | FNVIVMVLICFQAIAMMIDTDVQ-----SLQMSIALYWINSIFVMLYTMECILKLIAFRC |
| XP_010350732.1 Na_x_ IV *S. bol. boliviensis* | FNVIVMVLICFQAIAMMIDTDEH-----SLQMYIALYWINLIFVVLYTMECILKLIAFRC |
| XP_005315754.1 Na_x_ IV *I. tridecemlineatus* | FNIIIMVLIHVQAVTIMIQSDEQ-----SQQMDIAVYWINAIFVILYTLECILKLISFHC |
| XP_004857664.1 Na_x_ IV *Heterocephalus glaber* | FNAIIMVLICLQAMIMMIESDDQ-----SLQMDLALYWINATFVMLYTVECILKLISFHC |
| XP_005393341.1 Na_x_ IV *Chinchilla lanigera* | FNVSIMVLICCQAIAMMIENDEQ-----SLQVDTALHWVNSAFVVLYTVECILKLIAFHC |
| XP_008136835.1 Na_x_ IV *Eptesicus fuscus* | FNIIIMVLICFQAITLMIQSDEQ-----SRQMDMVVLWLQLLFVILYTGECVLKLIAFRC |
| XP_004577381.1 Na_x_ IV *Ochotona princeps* | FNVIIIVLICFQVITLMIQSDGQ-----SAQMQTAIYWINSIFVMLYTLECLLKLFSFVC |
| XP_004674928.1 Na_x_ IV *Condylura cristata* | FNIIVMIFICCQAIPIMLESDEQ-----SPNMDIALYWINLILLILYTGECVLKLIAFRC |
| XP_002923846.1 Na_x_ IV *Ailuropoda melanoleuca* | FNAIVMALICFQAITIMIQSDEQ-----SQNMDIALCWINLVLIILYTGECILKLIAFHC |
| XP_006736092.1 Na_x_ IV *Leptonychotes weddellii* | FNAIIIALICFQAITIMIQSDEQ-----SQNMDIALCWINLILIILYTGECVLKLIAFHC |
| XP_007074683.1 Na_x_ IV *Panthera tigris altaica* | FNVFIMVLICFQAMTIMIQSDEQ-----SSEMDTALYWITLFLVMVYTGECVLKLISFRC |
| XP_005907969.1 Na_x_ IV *Bos mutus* | FNIIIMTLICFQAITIMIQSEEQ-----SPKIDTALFWINSVYVMLYIGECVLKLIVFHC |
| XP_006180754.1 Na_x_ IV *Camelus ferus* | FNVIIMVLTCFQAITIMIQSDEQ-----SPKMDVALYWSHSVFVMLYTGECVLKLIAFHC |
| XP_007183279.1 Na_x_ IV *B. acu. Scammoni* | FDVIIMVLIFFQAITIMIQSDEQ-----NPKIDIALYWINSVFVILYTGECVLKLVVFHY |
| XP_001496911.1 Na_x_ IV *Equus caballus* | FNIIIMVLICFQAITIMIESDEQ-----SPKMDTALHWINAVFVMLYTGECILKLISFHC |
| XP_004428289.1 Na_x_ IV *C. simum simum* | FNIVIMVLICFQAITIMIQSDEQ-----NPKMDTALYWINSVFVMLYTGECVLKLISFPF |
| AEW90238.1 Na_v_2.5 IV *Hydra vulgaris* | FEFFIMSVILLNMVTMMLQHHHQ-----SLEFDLALIYLNYLFTGIFTLEAIVRLIAMRL |
| AAC38974.1 Na_v_ IV *Polyorchis penicillatus* | FEFFIMSVILLNMLTMMIQHHGQ-----SQQVSLALEYLNFLFTGIFTLEAIIRLTAMRL |
| AAA75572.1 Na_v_ IV *Cyanea capillata* | FELFIMSVIIANMLTMMIQHYDQ-----TKEVEVALNYLNYLFTGIFTIEAIIRLVAMRL |
| AEX00066.1 Na_v_2.2 IV *Trichoplax adhaerens* | FDLFIMTIIFANTITMMVNHYQE-----AKEVSDALRIIDIIFTSIYTLEAILKILAMRR |
| XP_028519297.1 Na_v_1.4 IV *Aiptasia pallida* | FETLIMFFICLNILVMMIQHYGQ-----KPAVEQALMIINLVFTGLFTLEAILRIVVLRL |
| AEX00073.1 Na_v_2.4 IV *Nematostella vectensis* | FEVFIMLFITINMVVMMVQHYDQ-----SKEVYDTLEILNLIFTSVFILEAILRIIALRK |
| AEW90239.1 Na_x_ IV *Clytia hemisphaerica* | FESFTMILIFVNIILMMFQHYQQ-----STDYDRMLYVTNLVFTVIFSIELILRIVALRQ |
| XP_002125324.3 Na_v_1.1 IV *Ciona intestinalis* | FELAVFLLILMNMITLMMEHYKM-----SYEWTVALKYADISFTALFSIEAGLKLIGMRY |
| XP_008161265.1 Na_v_1.5 IV *Chrysemys picta bellii* | FEITIVTLICLNMIIMMWVSEDQ-----DEKSKNILEIINKVFVAIFTGECIMKILALRY |
| DAA34928.1 Na_v_1.9 IV *Gallus gallus* | FDISVVTLICLNMVVMMAES--D-----NGEVRDVLKKINYFFVTVFTGECIIKIIALRH |
| XP_010200872.1 Na_v_1.5 IV *Colius striatus* | FDMTVVIFICLNMVVMMAEN--S-----QSGIKDVLNKINYFFVAVFTTECVIKILALRQ |
| XP_009068651.1 Na_v_1.5 IV *Acanthisitta chloris* | FDITIVIFICLNMVVMMAEN--N-----ENSIKPSLNKINYFFVVVFAAECVIKILGLRQ |
| ESO08974.1 Na_v_ IV *Helobdella robusta* | FDVAIMVVILLNMTTMGLEHYDQ-----PKAMEITLNYINTVFIVIFTLECVMKLLGLRF |
| BAA03398.1 Na_v_ IV *Heterololigo bleekeri* | FEIFITTIIITNMIFMAFEHYNQ-----SEVVTEVLATANIAFTILYAVEAIIKIIGLRI |
| EFX89321.1 Na_v_ IV *Daphnia pulex* | FEIAIFIMIFLNMVVMGVEHYGQ-----PPVFTFILELCNALFTTMFALEAVVKMIGLRH |
| AGL91670.1 Na_v_ IV *L. bostrychophila* | FEIAIFILIFLNMVTMAIEHYDQ-----PHAVFFILEVSNAFFTTVFGLEATVKIIGLRH |
| EEB12429.1 Na_v_1.2 IV *P. humanus corporis* | FEIAIFILIFLNMVTMAVEHYDQ-----PHAIFFVLEVSNAFFTTVFGLEALVKIVGLRY |
| XP_008196059.1 Na_v_ IV *Tribolium castaneum* | FEIAIFVLIFLNMLSMGIEHYNQ-----PHAVFFILEVSNAFFTTVFGLEAIVKIIGLRY |
| AFC61133.1 Na_v_1.1 IV *Bombyx mori* | LEIAIFVLIFLNMLTMGIEHYDQ-----PHSVFFILEVSNAFFTTVFGLEAIVKIVGLRY |
| EHJ64356.1 Na_v_1.1 IV *Danaus plexippus* | FEIAIFVLIFLNMLTMGIEHYDQ-----PHSVFFVLEVSNAFFTTVFGLEAIVKIIGLRY |
| XP_004535967.1 Na_v_ IV *Ceratitis capitata* | FEIAIFVLIFLNMLTMGIEHYNQ-----PHSVFFILEVSNAFFTTVFGLEAIVKIIGLRY |
| KFB48601.1 Na_v_ IV *Anopheles sinensis* | FEIAIFVLIFLNMLTMGIEHYDQ-----PHAVFFVLEVSNAFFTTVFGLEAIVKIVGLRY |
| EAA04706.4 Na_v_ IV *Anopheles gambiae* | FEIAIFVLIFLNMLTMGIEHYDQ-----PHAVFFVLEVSNAFFTTVFGLEAIVKIVGLRY |
| AFH08262.1 Na_v_ IV *Drosophila melanogaster* | FEIAIFVLIFLNMLTMGIEHYDQ-----PHAVFFILEVSNAFFTTVFGLEAIVKIVGLRY |
| EDW72405.1 Na_v_ IV *Drosophila willistoni* | FEIAIFVLIFLNMLTMGIEHYDQ-----PHAVFFILEVSNAFFTTVFGLEAIVKIVGLRY |
| EDV37233.1 Na_v_ IV *Drosophila ananassae* | FEIAIFVLIFLNMLTMGIEHYDQ-----PHAVFFILEVSNAFFTTVFGLEAIVKIVGLRY |
| XP_005178206.1 Na_v_ IV *Musca domestica* | ---AIFVLIFLNMLTMGIEHYNQ-----PHAVFFILEVSNAFFTTVFGLEAIVKIVGLRY |
| XP_003704512.1 Na_v_ IV *Megachile rotundata* | FEIAIFILIFLNMLTMGIEHYGQ-----PHLIFFLLYVSNAFFTTVFGLEAIVKIIGLRY |
| AFC61134.1 Na_v_1.1 IV *Nilaparvata lugens* | FEIAIFVLIFLNMLTMGIEHYNQ-----HHAIFFVLEVSNAFFTTVFGLEALVKIVGLRY |
| AAK01090.1 Na_v_1.1 IV *Blattella germanica* | FEIAIFVLIFLNMLTMGIEHYQQ-----PHAVFFILEVSNAFFTTVFGLEAIVKIIGLRY |
| XP_003697620.1 Na_v_ IV *Apis florea* | FEIAIFILIFLNMLTMGIEHYDQ-----PHPIFFVLEVSNAFFTTVFGLEAIVKIIGLRY |
| XP_003493336.1 Na_v_ IV *Bombus impatiens* | FEIAIFILIFLNMLTMGIEHYDQ-----PHPIFFVLEVSNAFFTTVFGLEAIVKIIGLRY |
| EZA62325.1 Na_v_ IV *Cerapachys biroi* | FEIAIFVLIFLNMLTMGIEHYNQ-----PHAIFFVLEVSNAFFTTVFGLEAIVKIIGLRY |
| XP_008545189.1 Na_v_ IV *Microplitis demolitor* | FEIAIFVLIFLNMLTMGIEHYNQ-----PHPIFFILEVSNAFFTTVFGLEAIVKIIGLRY |
| XP_793384.3 Na_v_ IV *S. purpuratus* | FELVIVTVIVMNMILMMIDHYGM-----SQELTDITAKFNIVFTSIFVCEAVLKLIGYSW |
| EEN42493.1 Na_v_ IV *Branchiostoma floridae* | FDITIMVFICLNMVSMMIEHYRQ-----SDKLKEALELINIIFIAVFTFEAVLKIIGQRW |
| EKC21550.1 Na_v_1.5 IV *Crassostrea gigas* | FDLCIVIVIFLNMIAMAVDHYKM-----TDYVSNILDILNILFTTIFTLECVIKIIGLRH |
| ESO94539.1 Na_v_ IV *Lottia gigantea* | FELSIIVIIFLNMITLAIESYKQ-----SDTIRDALKILNIIFTVIFILECMIKLIGMRW |
| ELT91324.1 Na_v_ IV *Capitella teleta* | FEFAIVVLIFLNMISMAQEHYQQ-----SQAYTDTLDIMNIIFTTIFTLEALVKIFGMRW |
| XP_006818874.1 Na_v_ IV *Saccoglossus kowalevskii* | FEISIVLLICLNMVAMAVEHYEQ-----SQQFSDVLDAINIVFVAIFTLEAIMKIIGMRW |
| XP_007059504.1 Na_v_1.5 IV *Chelonia mydas* | FDVIIMILICLNMITMMVETDDQ-----STEKTYILNKINMLFVAIFTAECIMKLLALRH |
| XP_009941400.1 Na_v_1.5 IV *Opisthocomus hoazin* | FDVVIMILICLNMIAMMVETHGQ-----SEMKTNILEKINILFVAVFTAECVLKLVALRQ |
| XP_010158440.1 Na_v_1.5 IV *Eurypyga helias* | FDVVIMILICLNMIAMMVETYDQ-----SPVKANILNKINIFFIAIFTAECGLKLVALRQ |
| XP_009084518.1 Na_v_1.5 IV *Serinus canaria* | FDVVIMGLICLNMVTMMVETYEQ-----SETKTNVLSKINILFVTIFTAECVLKLLALRQ |
| XP_008920014.1 Na_v_1.5 IV *Manacus vitellinus* | FDVVIMVLICLNMIMMMVETYEQ-----SETKTIVLNKINILFVVIFTAECVLKLVALRQ |
| XP_005512551.1 Na_v_1.5 IV *Columba livia* | FDIVIMVLICLNMVSMMVETYDQ-----SLTKTEVLNKINILFVTVFTAECVLKLVALRQ |
| XP_009286087.1 Na_v_1.5 IV *Aptenodytes forsteri* | FDVVIMILICLNMITMMVETHEQ-----SQTKTNILSKINILFIAIFTAECVLKLVALRQ |
| XP_009642396.1 Na_v_1.5 IV *Egretta garzetta* | FDVVIMTLICLNMVTMMVETYDQ-----SRTKANVLDKINKLFVAIFTAECVLKLVALRQ |
| XP_003206989.1 Na_v_1.5 IV *Meleagris gallopavo* | FDIAIMILICLNMITMMVETYEQ-----SDTKTNVLNKINILFVAIFTTECILKLVALRQ |
| XP_009962499.1 Na_v_1.5 IV *Tyto alba* | FDVVIMILICLNMITMMVETYEQ-----SATKTNVLNQINILFIAIFTAECVLKLVALRQ |
| XP_009479426.1 Na_v_1.5 IV *Pelecanus crispus* | FDVVIMILICLNMITMMVETYEQ-----SQTKTNILDKINKLFVAIFTAECVVKLVALRQ |
| XP_009465855.1 Na_v_1.5 IV *Nipponia nippon* | FDIVVMILICLNMITMMVETYEQ-----SNTKTNILNKINILFVAIFTAECALKLVALRQ |
| XP_007565048.1 Na_v_1.4 IV *Poecilia formosa* | FDMLIMVLIGLYTLMMMVETDEQ-----SPKMEEILYWVNLVFIMIFSIECCLKMIALRK |
| CBY22707.1 Na_v_ IV *Oikopleura dioica* | FEITIMILILSNMITMLVEHEEM-----SPNFESVLEFINYIFIAIFTGECVLKMFALRH |
| XP_008111415.1 Na_v_1.4 IV *Anolis carolinensis* | FDIVIMILICLNMVTMMVETDDQ-----SQTKIDILFQINLIFIVIFTTECFLKMIALRY |
| AAW68223.1 Na_v_1.4 IV *Thamnophis sirtalis* | FDIIIMILICLNMVTMMVETDDQ-----SQTKITILAQINLVFIIIFTSECFLKMIALRH |
| XP_007424896.1 Na_v_1.4 IV *Python bivittatus* | FDIVIMILICLNMVTMMVETDDQ-----SQTKINILAQINLIFIIIFTSECFLKMIALRY |
| XP_007628932.1 Na_v_1.4 IV *Cricetulus griseus* | FDISIMILICLNMVTMMVETDDQ-----SQLKVDILYNINMVFIIIFTGECVLKMFALRH |
| XP_003768502.1 Na_v_1.4 IV *Sarcophilus harrisii* | FDIMIMFLICLNMVTMMVETDDQ-----SQLKIDILYNINMVFIIIFTGECVLKMVALRH |
| XP_006274845.1 Na_v_1.4 IV *A. mississippiensis* | FDITIMVLICLNMVTMMVETDDQ-----SEAKIEILQTVNLIFIIIFTGECVLKMFALRY |
| XP_005283172.1 Na_v_1.4 IV *Chrysemys picta bellii* | FDIIIMVLICLNMVTMMVETDDQ-----SPTKINVLYNINMIFIVIFTGECLLKMFALRY |
| XP_002933087.2 Na_v_1.4 IV *Xenopus tropicalis* | FDIVIMILICLNMVTMMIETDDQ-----SQEKTDILFKINLVFIVIFTAECVLKMFALRY |
| XP_010018959.1 Na_v_1.4 IV *Nestor notabilis* | FDIVIMILICLNMVTMMVETDDQ-----SELKTSVLYKINLVFIVIFTGECVLKMFALRH |
| XP_009683652.1 Na_v_1.4 IV *S. camelus australis* | FDITIMILICLNMVTMMVETDDQ-----SQFKTDVLYKVNLVFIVIFTGECVLKMFALRY |
| XP_010226082.1 Na_v_1.4 IV *Tinamus guttatus* | FDITIMILICLNMVTMMVETDDQ-----SQLKTDVLYKVNLVFIVIFTGECVLKMFALRY |
| XP_009073953.1 Na_v_1.4 IV *Acanthisitta chloris* | FDITIMILICLNMVTMMVETDDQ-----SELKTSVLYQINLVFIVVFTGECVLKMFALRY |
| DAA34926.1 Na_v_1.4 IV *Gallus gallus* | FDITIMILICLNMVTMMVETDDQ-----SETKTDILYKINLIFIVIFTGECVLKMFALRY |
| KFO60281.1 Na_v_1.2 IV *Corvus brachyrhynchos* | --ITIMILICLNMVTMMVETDDQ-----SELKTSVLYKINLVFIVIFTGECVLKMFALRY |
| XP_008931935.1 Na_v_1.4 IV *Manacus vitellinus* | FDITIMILICLNMVTMMVETDDQ-----SELKTSVLYKINLVFIVIFTGECVLKMFALHY |
| XP_010131382.1 Na_v_1.4 IV *B. rhinoceros silvestris* | FDITIMILICLNMVTMMVETDDQ-----SEFKTSILNKINLVFIVIFTGECVLKMFALRY |
| XP_005021806.1 Na_v_1.4 IV *Anas platyrhynchos* | FDITIMILICLNMVTMMVETDDQ-----SELKTDILYKINLVFIVIFTGECVLKMFALRY |
| KFP89920.1 Na_v_1.2 IV *Apaloderma vittatum* | FDITIMILICLNMVTMMVETDDQ-----SELKTSVLYKINLVFIVIFTGECVLKMFALRY |
| XP_005508265.1 Na_v_1.4 IV *Columba livia* | FDITIMILICLNMVTMMVETDDQ-----SELKTSILYKINLVFIVIFTGECVLKMFALRY |
| XP_009997626.1 Na_v_1.4 IV *Chaetura pelagica* | FDITIMILICLNMVTMMVETDDQ-----SELKTSVLYKINLVFIVIFTGECVLKMFALRH |
| XP_009565587.1 Na_v_1.4 IV *Cuculus canorus* | FDITIMILICLNMVTMMVETDDQ-----SELKTSILYKINLVFIVIFTGECVLKMFALRY |
| KFQ96635.1 Na_v_1.2 IV *Nipponia nippon* | FDITIMILICLNMVTMMVETDDQ-----SELKTSVLYKINLVFIVIFTGECVLKMFALRY |
| XP_009700369.1 Na_v_1.4 IV *Cariama cristata* | FDITIMILICLNMVTMMVETDDQ-----SELKTSVLYKINLVFIVIFTGECVLKMFALRY |
| KFQ13298.1 Na_v_1.5 IV *Leptosomus discolor* | FDITIMILICLNMVTMMVETDDQ-----SELKTSVLYKINLVFIVIFTGECVLKMFALRY |
| KFP55758.1 Na_v_1.2 IV *Cathartes aura* | FDITIMILICLNMVTMMVETDDQ-----SELKTSVLYKINLVFIVIFTGECVLKMFALRY |
| XP_004081947.1 Na_v_1.2 IV *Oryzias latipes* | FDIVIMVLIWLNMVTMMVETADQ-----SEKQTYILRVINYVFIVIFSGECLLKMIGLRH |
| XP_005986260.1 Na_v_1.6 IV *Latimeria chalumnae* | FDICIMILICLNMVTMMVETDDQ-----SDQTEVVLYWVNFIFIIVFTSECVLKLFALRH |
| XP_005430143.1 Na_v_1.6 IV *Geospiza fortis* | FDIVIMMLICLNMVTMMVETDTQ-----SKQMEDILYWINFVFVIFFTCECVLKMFALRH |
| XP_002939316.2 Na_v_1.2 IV *Xenopus tropicalis* | FDIIIMILIFLNMVTMMIETDDQ-----SQEMEMYLYRINAVFIILFTGEFLLKLISLRQ |
| XP_005519819.1 Na_v_1.2 IV *Pseudopodoces humilis* | FDITVMVLICLNMVTMMIETDDQ-----TELMQNILYWINLVFVVLFTGECVFKIFSLRY |
| XP_009086199.1 Na_v_1.2 IV *Serinus canaria* | FDIIIMVLICLNMVTMMIETDDQ-----NELMQNILYWINLVFVVLFTGECVFKIFSLRY |
| XP_010121454.1 Na_v_1.2 IV *Chlamydotis macqueenii* | FDITIMVLICLNMVTMMIETDDQ-----GELMKSILYWINLVFVVLFTGECVLKLFSLRY |
| XP_009553849.1 Na_v_1.2 IV *Cuculus canorus* | FDITIMVLICLNMVTMMIETDDQ-----GQLMQDILYWINLVFVVLFTGECILKMFSLRY |
| XP_009892890.1 Na_v_1.2 IV *Charadrius vociferus* | FDITIMVLICLNMVTMMIETDDQ-----SELMQNILYWINLVFVVLFTGECVFKMFSLRY |
| XP_009639044.1 Na_v_1.2 IV *Egretta garzetta* | FDITIMVLICLNMVTMMIETDDQ-----GELMQNILYWINLVFVVLFTGECVFKLFSLRY |
| XP_009994594.1 Na_v_1.2 IV *Chaetura pelagica* | FDITIMVLICLNMVTMMIETDDQ-----GELMQNILYWINLVFVVLFTGECILKLFSLRY |
| DAA34919.1 Na_v_1.6 IV *Xenopus tropicalis* | FDIVIMILICLNMVTMMVETDDQ-----SDYTDNVLYWINVVFIVFFTTECVLKLCALRH |
| ELR47486.1 Na_v_1.7 IV *Bos mutus* | FDIAIMVLICLNMVTMMVEKEGQ-----SDYVTEVLNWINVVFIILFSGECVLKLISLRC |
| XP_004322655.1 Na_v_1.7 IV *Tursiops truncatus* | FDIAIMVLICLNMVTMMVEKEGQ-----SAYMTEVLYWINVVFVILFTGECVLKLISLRC |
| XP_003795197.1 Na_v_1.7 IV *Otolemur garnettii* | FDITIMVLICLNMVTMMVEKEDQ-----SDEMTNVLYWINVVFITLFTGECVLKLISLRH |
| XP_009441903.1 Na_v_1.7 IV *Pan troglodytes* | FDISIMVLICLNMVTMMVEKEGQ-----SQHMTEVLYWINVVFIILFTGECVLKLISLRH |
| AAA89159.1 Na_v_ IV *Oryctolagus cuniculus* | FDITIMILICLNMVTMMVEKEGQ-----SDYMTDVLYWINVVFIILFTGECVLKLISLRH |
| XP_004577152.1 Na_v_1.7 IV *Ochotona princeps* | FDISIMVLICLNMVTMMVEKDDQ-----SVYMTHVLYWINVVFIILFTGECVLKLISLRH |
| XP_006180752.1 Na_v_1.7 IV *Camelus ferus* | FDITIMVLICLNMVTMMVEKEGQ-----SEYMTEVLYWINVVFIILFTGEFVLKLISLRC |
| XP_007945734.1 Na_v_1.7 IV *Orycteropus afer afer* | FDITIMVLICLNMVTMMVEKEGQ-----SKYMTDVLQWINVVFIILFTGECVLKLISLRH |
| XP_006921216.1 Na_v_1.7 IV *Pteropus alecto* | FDITIMVLICLNMVTMMVEKEGQ-----SAYMTNVLYWINVVFIILFTGECVLKLISLRH |
| XP_008829101.1 Na_v_1.7 IV *Nannospalax galili* | FDITIMVLICLNMVTMMVEKEGQ-----SDYMTKILYWINVVFIILFTGECVLKLISLRH |
| XP_004674637.1 Na_v_1.7 IV *Condylura cristata* | FDITIMVLICLNMVTMMVEKEGQ-----STYMTDVLYWINVVFIILFTGECVLKLISLRH |
| XP_006879389.1 Na_v_1.7 IV *Elephantulus edwardii* | FDITIMVLICLNMVTMMVEKEGQ-----SDYMTDVLYWINVVFIILFTGECVLKLISLRH |
| XP_006003324.1 Na_v_1.4 IV *Latimeria chalumnae* | FDIFIMVLICLNMVTMMIETDDQ-----SAEKEEILYWVNLVFIVVFTGECILKIIALRH |
| XP_006003661.1 Na_v_1.2 IV *Latimeria chalumnae* | FDIIIMILICLNMVTMMVETDNQ-----SKEMEKNLYWVNLVFIVLFTGECILKLISLRH |
| XP_006636602.1 Na_v_1.2 IV *Lepisosteus oculatus* | FDITIMILICLNMVTMMVETDDQ-----SKDMENILYWINLVFIVLFTGECVLKLISLRH |
| XP_008113359.1 Na_v_1.2 IV *Anolis carolinensis* | FDISIMILICLNMVTMMVETDDQ-----TDAMETNLYRINLIFIVLFTGECVLKLISLRY |
| XP_006137390.1 Na_v_1.3 IV *Pelodiscus sinensis* | FDIGIMILICLNMVTMMIETDDQ-----SDEMETILQRINLVFIVLFTGECVLKLISLRY |
| XP_008997034.1 Na_v_1.3 IV *Callithrix jacchus* | FDISIMILICLNMVTMMVETDDQ-----GKYMTLVLSRINLVFIVLFTGEFVLKLVSLRH |
| XP_006160314.1 Na_v_1.3 IV *Tupaia chinensis* | FDISIMILICLNMVTMMVETDDQ-----SKYMTLILSRINLVFIILFTGEFVLKLISLRY |
| XP_004601166.1 Na_v_1.3 IV *Sorex araneus* | FDISIMILICLNMVTMMVETDDQ-----SKYMTLVLSRINLVFIILFTGEFVLKLISLRY |
| XP_009674477.1 Na_v_1.2 IV *S. camelus australis* | FDISIMILICLNMVTMMVETDDQ-----SKEMETILSRINLVFIILFTGECVLKLISLRH |
| XP_001367154.1 Na_v_1.3 IV *Monodelphis domestica* | FDISIMILICLNMVTMMVETDDQ-----SDDMTSILSRINLIFIVLFTGECVLKMISLRH |
| XP_008136988.1 Na_v_1.1 IV *Eptesicus fuscus* | FDISIMILICLNMVTMMVETDDQ-----SEYVTSVLARINLVFIVLFTGECVLKLISLRH |
| XP_003795205.1 Na_v_1.1 IV *Otolemur garnettii* | FDISIMILICLNMVTMMVETDDQ-----SEYVTTILSRINLVFIVLFTGECVLKMISLRH |
| XP_005946612.1 Na_v_1.5 IV *Haplochromis burtoni* | FDIIIMVLILFNMITMMVETDEQ-----PPQMEKILNNINLAFIIIFTAECLIKIMALRC |
| XP_007233940.1 Na_v_1.2 IV *Astyanax mexicanus* | FDIMIMLLILLNMVTMMVETDEQ-----SPRMEMILYNINLAFIVIFTTECIIKLIALRC |
| ABA54922.1 Na_v_1.5 IV *Danio rerio* | FDITIMMLIILNMITMMVETDEQ-----SARMETILNNINLAFIVIFTTECLIKIFALRC |
| XP_005475994.1 Na_v_1.4 IV *Oreochromis niloticus* | FDIMIMMLIIVNMVTMMVETDEQ-----SERMESILNKINLVFIVIFTTECLIKIFALRC |
| XP_005813731.1 Na_v_1.4 IV *Xiphophorus maculatus* | FDIMIMMLIIVNMVTMMVETDEQ-----SERMESVLNIINLVFIVIFTTECLIKLFALRC |
| XP_007241580.1 Na_v_1.4 IV *Astyanax mexicanus* | FDILIMVLICLNMITMMAETDEQ-----SIEVEEILFYINFAFIVIFTGECVLKIIALRQ |
| ABB29444.1 Na_v_1.4 IV *Tetraodon nigroviridis* | FDVFIMVLICLNMVTMMVETDEQ-----TKEKEDILYWINVIFIVIFTTECILKTIALRR |
| ABB29442.1 Na_v_1.4 IV *Takifugu rubripes* | FDVFIMVLICLNMVTMMVETDEQ-----SDKKEEVLYWINVVFILIFTTECTLKIIALRR |
| XP_002125324.3 Na_v_1.1 II *Ciona intestinalis* | FETVIMLSIIINTLFLAIDHHGI-----SPELNNALNVGNHVFTGIFTVEAIIKITALGV |
| ELT91324.1 Na_v_ II *Capitella teleta* | FELFITACIVLNVIVMALEFHGM-----SEELRSGLQVANYVFSAIFIVEAIVKLLALSK |
| EKC21550.1 Na_v_1.5 II *Crassostrea gigas* | FDLFITFCILINTIFMGIEYHNM-----PQGLVDATTWANFVFTIIFTLEAVLKLCAFGK |
| XP_006818874.1 Na_v_ II *Saccoglossus kowalevskii* | VDVFITLCILVNTLFLAMDHYNM-----PETWEKSLDYGNKVFTGIFVLEATMKIIALDA |
| BAA03398.1 Na_v_ II *Heterololigo bleekeri* | TEVFIIFIIVLNTVFLAMEHHGM-----SMELKNVLKVANYVFTTVFVLEAILKLLAFNK |
| ESO94539.1 Na_v_ II *Lottia gigantea* | LDLFITLCILMNTIIMACESHEM-----SETTQETIRISNYVFTSVFTLEAILKIIALSK |
| XP_793384.3 Na_v_ II *S. purpuratus* | MDLFITLCILGNTAFLMMDHEEI-----SDSLAYISEEGNKVFTYIFTIECVLKLIALDK |
| EFX89321.1 Na_v_ II *Daphnia pulex* | IDFVITISIVLNTAFLAAEHHGM-----SPDVKHVLDVGNKVFTSVFTTECILKMGATGT |
| KFB48601.1 Na_v_ II *Anopheles sinensis* | FELGITLCIVLNTMFLALEHHGM-----NANVRDALDIGNKVFTSIFTLECILKVMALSK |
| EAA04706.4 Na_v_ II *Anopheles gambiae* | FELGITLCIVLNTMFLALEHHGM-----NANVRDALDIGNKVFTSIFTLECILKVMALSK |
| EHJ64356.1 Na_v_1.1 II *Danaus plexippus* | FELFITTCIVLNTLFLALEHHGM-----SENVRQALDIGNKVFTSIFTLECIMKVMAMSK |
| AFC61133.1 Na_v_1.1 II *Bombyx mori* | FELFITTCIVLNTLFLALEHHGM-----SENVRRVLDIGNKVFTSIFTLECIMKVMAMSK |
| EZA62325.1 Na_v_ II *Cerapachys biroi* | FELMITLCILLNTVFLALEHHGM-----SESIRQALNIGNKVFTSIFTFECFLKLLALSK |
| XP_003704512.1 Na_v_ II *Megachile rotundata* | FELTITLCIVLNTGFLAMEHHGM-----SESIRQALNIGNKVFTSIFTFECLLKLLALSK |
| XP_003493336.1 Na_v_ II *Bombus impatiens* | FELTITLCIVLNTGFLAMEHHGM-----SESIRQALNIGNKVFTSIFTFECLLKLLALSK |
| XP_003697620.1 Na_v_ II *Apis florea* | FELTITLCIVLNTGFLAMEHHGM-----SESIRQALNIGNKVFTSIFTFECLLKLLALSK |
| XP_008545189.1 Na_v_ II *Microplitis demolitor* | FELTITLCIVLNTGFLAMEHHGM-----SENVRQALNIGNKVFTSIFTFECFLKLLALSK |
| AAK01090.1 Na_v_1.1 II *Blattella germanica* | FDLLITLCIILNTMFLAMEHHGM-----SESVRQALDIGNKVFTSIFTLECFLKILALSK |
| EDV37233.1 Na_v_ II *Drosophila ananassae* | FELAITLCIVLNTAFLAMEHHGM-----SESFRNALDVGNKVFTSIFTFECIVKLMALSK |
| AFH08262.1 Na_v_ II *Drosophila melanogaster* | FELAITLCIVLNTAFLAMEHHGM-----SESFRNALDVGNKVFTSIFTFECIVKLMALSK |
| EDW72405.1 Na_v_ II *Drosophila willistoni* | FELAITLCIVLNTAFLAMEHHGM-----TESFRNALDVGNKVFTSIFTFECIVKLMALSK |
| XP_005178206.1 Na_v_ II *Musca domestica* | ---AITLCIVLNTAFLAMEHHGM-----SESFRNALDVGNKVFTSIFTFECIIKLMALSK |
| XP_004535967.1 Na_v_ II *Ceratitis capitata* | FELAITLCIVLNTAFLAMEHHGM-----SENFRNALDVGNKVFTSIFTFECIIKLMALSK |
| XP_008196059.1 Na_v_ II *Tribolium castaneum* | FELAITVCIVLNTMFLAMEHHGM-----SDDVLKALDIGNKVFTSIFTFECCLKLMALSK |
| AFC61134.1 Na_v_1.1 II *Nilaparvata lugens* | FELLITLCIVLNTMFLATEHHGM-----SESILNMLDIGNKVFTSIFTLECTLKLMALSK |
| AGL91670.1 Na_v_ II *L. bostrychophila* | FELLIAICIVLNTMFLAMEHHGM-----SENVRQALDIGNKVFTSIFSLECFLKLLALSK |
| EEB12429.1 Na_v_1.2 II *P. humanus corporis* | FELLITVCIVLNTMFLAMEHHGM-----SDSVRQALDIGNKVFTSIFTFECFLKLLALSK |
| AEX00073.1 Na_v_2.4 II *Nematostella vectensis* | MEIFIIVCILLNTLVMSIEHPRL-----EDPLLTVVNI-STVFTFIFLLEMILKLIALGF |
| XP_028519297.1 Na_v_1.4 II *Aiptasia pallida* | MDTFITFCTMVNTLFLSLEYHNM-----DSNYLMVLEIGNKVFTMVFLLEMILKITAFGF |
| AEX00066.1 Na_v_2.2 II *Trichoplax adhaerens* | FDLIITFCIALNTLFMALYQPNPRNDPGKASLNNVIQIANYVFTGIFTLEMVLKLIAFTP |
| AEW90239.1 Na_x_ II *Clytia hemisphaerica* | FELAVTIFILLNTVCLAIEHHNM-----DKNIEFVLQIANHVFTGIFILEMVLKLIALGL |
| AAA75572.1 Na_v_ II *Cyanea capillata* | FEGFITGCIMVNTIAMAAEHFEQ-----PEVMTKMSDILNYIFTSIFVIEMTCKLIALTP |
| AAC38974.1 Na_v_ II *Polyorchis penicillatus* | FEGFITFCIMLNTCLMASEHYKM-----PKKLEDALNVFNYIFTATFCIEMGFKILAFTP |
| AEW90238.1 Na_v_2.5 II *Hydra vulgaris* | FEGFITACIMLNTLLMALEHHNM-----SAKLTKITEIFNYVFTGIFIFEMMIKLVGYTP |
| CBY22707.1 Na_v_ II *Oikopleura dioica* | LDLFITLCIIANTVFMGLEKRPM-----EEDFEKMLQDANTIFTLIFALEMVIKLIGMHP |
| XP_008161265.1 Na_v_1.5 II *Chrysemys picta bellii* | TDLTITVCIVVNTIFMALEHDNM-----TETLSNMIITVNNVFTGIFTAEMIFKIIALDP |
| XP_009068651.1 Na_v_1.5 II *Acanthisitta chloris* | VDLLIMVCIVLNTLAMAMEYPGM-----PPNYQIMIYRSDKVFTLIFTVEMILKIIALDP |
| XP_010200872.1 Na_v_1.5 II *Colius striatus* | VDLLIMVCIVINTLFMALEHPGM-----DRSYRKMISRSDKVFTLIFAAEMILKIIALDP |
| DAA34928.1 Na_v_1.9 II *Gallus gallus* | VDLIIMICIIVNTIFMALEHPGM-----TSTEKRLICISDKVFTMVFAAEMVLKIIALDP |
| XP_007565048.1 Na_v_1.4 II *Poecilia formosa* | VDITII---VLYIILMATEHYPM-----TPEFYELINTGNLVLTGILIAEMVLKILAMDP |
| XP_003766750.1 Na_v_1.7 II *Sarcophilus harrisii* | FDLAIVICVIMNLIFLAMDHYPM-----TEEFLYVLFVGHQVFTGIYVLEMILKIIALHP |
| XP_004857664.1 Na_x_ II *Heterocephalus glaber* | TDLFIFICIILNTHILALEHYPM-----SEDTSYFLSIGNIACIGIFTAEMILKIIAMHP |
| XP_008256919.1 Na_x_ II *Oryctolagus cuniculus* | TDLFFTMCIILNIYFLALEHYPM-----SDETNHLLIIGNLVFIGIFTAEMIFKIIAMHP |
| XP_005393341.1 Na_x_ II *Chinchilla lanigera* | TDLFLTICIILNIFILAMEHYPM-----SEDTWSLLNIGNMIFVGIFTAEMILKIIAMHP |
| XP_002812597.1 Na_x_ II *Pongo abelii* | TDLFFIICIILNVCFLALEHYPM-----SKQTDNLLNIGNLVFIGIFKAEMIFKIIAMHP |
| XP_006153638.1 Na_x_ II *Tupaia chinensis* | TDLFLTICIILNTCFLAVEHYPM-----GDETINLLSIGNLVFLGIFTAEMIFKIIAMHP |
| XP_010363599.1 Na_x_ II *Rhinopithecus roxellana* | TDLFLIICIILNICFLALEHYPM-----SKSTNNLLNIGNLVFIGIFTAEMIFKIIAMHP |
| XP_008582173.1 Na_x_ II *Galeopterus variegatus* | TDLFLIICIILNIHFLAIEHYPM-----SEETNNLLSIGNLVFIGIFTAEMILKIIVMHP |
| XP_010350732.1 Na_x_ II *S. bol. boliviensis* | TDLFLFICIILNVCFLALEHYPM-----SEQTSNILSIGNLVFIGIFTAEMILKIIAMHP |
| XP_006867096.1 Na_x_ II *Chrysochloris asiatica* | TDLVIIVCIILNIIFLALEHYPM-----TVEISTLLSIGNLVFIGIFTAEMIFKIIAMHP |
| XP_007539379.1 Na_x_ II *Erinaceus europaeus* | ADLFLVMCIILNIYFLALEHYPM-----TVETSNVLSIGNLVFIGIFTAEMIFKVIAMHP |
| XP_007945562.1 Na_x_ II *Orycteropus afer afer* | TDLALIICIILNTLFLALQYYPL-----SVKMDNVLAIGNMVFIGIFTAEMIFKIIAMHP |
| XP_004577381.1 Na_x_ II *Ochotona princeps* | TDLFLTMCIILSIYFLALEHYPM-----SEGISQLLSLGNLVIIAIFTIEMIFKIIAMHP |
| XP_005315754.1 Na_x_ II *I. tridecemlineatus* | TDLFLLICIILNIYFLAWEHYPM-----SDDHSYLLSIGNLVFLGIFTAEMIFKIIAMNP |
| XP_003405861.1 Na_x_ II *Loxodonta Africana* | TDLALAICIILNILFLALEHYPR-----SEGTNSLLSIGNLVFIGIFTAEMILKIIAMHP |
| XP_004674928.1 Na_x_ II *Condylura cristata* | TDLFLTICIIANIYFLALEHYPM-----SKETSSVLSIGNLVFIGIFTTEMILKIIAMHP |
| XP_002923846.1 Na_x_ II *Ailuropoda melanoleuca* | TDLFLTICIILNICFLALECYPM-----SEETNNSLSIGNLIFIGIFTAEMSLKIIAMHP |
| XP_006736092.1 Na_x_ II *Leptonychotes weddellii* | TDLVLTVCIILNICFLALECYPM-----SEETNNILSIGNLIFIGIFAAETILKITAMHP |
| XP_007074683.1 Na_x_ II *Panthera tigris altaica* | TDLVLTICIILNVCFLALECYPM-----SEETTSVLSIGNLIFIGIFTAEMILKIIAMHP |
| XP_008136835.1 Na_x_ II *Eptesicus fuscus* | TDLFLTICIILNIYFLAMECYPM-----SAATSNVLSIGNLVFIGIFTTEMIFKIIAMHP |
| XP_001496911.1 Na_x_ II *Equus caballus* | TDLILTICIILNMYFLALEHYPM-----SEETISVLSIGNLVFLGIFTAEMIFKIIAMHP |
| XP_004428289.1 Na_x_ II *C. simum simum* | TDLILTICIILNMYFLALEHYPM-----SEETISILSIGNLVFIGIFTAEMIFKIIAMHP |
| XP_005907969.1 Na_x_ II *Bos mutus* | TDLFLTICIILNIHFLALEHYPM-----SLETSNILSIGNVVFIGIFTAEMIFKIIAMYP |
| XP_006180754.1 Na_x_ II *Camelus ferus* | TDLFLTICIILNIQFLALEHYPM-----SAETNNVLSIGNLVFIGIFTTEMIFKIIAMHP |
| XP_007183279.1 Na_x_ II *B. acu. Scammoni* | TDLFLTICIILNIHFLALEHYPM-----SLETNNILNIENQVFIGIFTAEMIFKIIAMHP |
| ESO08974.1 Na_v_ II *Helobdella robusta* | VDLFITLCIVVNTVFMAMEHANM-----HDTLVKVLSYGNYVFTGIFTVEAALKILALGL |
| XP_007059504.1 Na_v_1.5 II *Chelonia mydas* | TDLAITVCIVMNTIFMALEHHNM-----TNTFKFMLRVGNMVFTGIFTAEMILKIIALDP |
| XP_008920014.1 Na_v_1.5 II *Manacus vitellinus* | FDLAITVCIVMNTLFMALEHNDM-----SHTFKFMLKVGNLVFTGIFTAEMILKIIALDP |
| XP_005512551.1 Na_v_1.5 II *Columba livia* | IDLTITVCIVVNTLFMALEHNNM-----SEEFKFMLKVGNLVFTGIFTAEMVLKIIALDP |
| XP_009084518.1 Na_v_1.5 II *Serinus canaria* | FDLTITVCIVMNTLFMALEHNNM-----SPTFKFMLKIGNLVFTGIFTAEMILKIIALDP |
| XP_003206989.1 Na_v_1.5 II *Meleagris gallopavo* | IDLTITLCIVMNTLFMALEHNNM-----THNFKLMLSVGNSIFTGIFTAEMVLKIIALDP |
| XP_009286087.1 Na_v_1.5 II *Aptenodytes forsteri* | VDLTITVCIVMNTLFMALEHNNM-----SDNFGWMLNVGNLIFTGIFTTEMILKIIALDP |
| XP_009642396.1 Na_v_1.5 II *Egretta garzetta* | IDLTITVCIVVNTLFMALEHNNM-----SANFKFMLNVGNLVFTGIFTAEMILKIIALDP |
| XP_010158440.1 Na_v_1.5 II *Eurypyga helias* | IDLTITLCIVMNTLFMALEHNNM-----SDNFKSMLNVGNLVFTGIFTAEMILKIIALDP |
| XP_009479426.1 Na_v_1.5 II *Pelecanus crispus* | IDLTITVCIVMNTLFMALEHDNM-----TDNFKLMLNVGNLVFTGIFTAEMILKIIALDP |
| XP_009465855.1 Na_v_1.5 II *Nipponia nippon* | IDLTITVCIVMNTLFMALEHNNM-----SDNFKLMLNVGNLVFTGIFTAEMILKIIALDP |
| XP_009962499.1 Na_v_1.5 II *Tyto alba* | IDLTITVCIVMNTLFMALEHNNM-----SENFKSMLNVGNLVFTGIFTAEMILKIIALDP |
| XP_009941400.1 Na_v_1.5 II *Opisthocomus hoazin* | IDLLITVCIVMNTLFMALEHNNM-----SENFKSMLNVGNLVFTGIFTAEMILKIIALDP |
| EEN42493.1 Na_v_ II *Branchiostoma floridae* | IDLLITLCIVLNTMFMAMDHYGK-----SESFELTLKTGNYVFTAIFAAEFFLKLLALGP |
| XP_004081947.1 Na_v_1.2 II *Oryzias latipes* | ADLTITICIVLNTLFMAMEHHPM-----QDEFTKMLSIGNKVFTGIFTAEMVLKIIALDP |
| XP_002939316.2 Na_v_1.2 II *Xenopus tropicalis* | VDLAITISIVLNTIFMAVEHAHM-----TPYFISVLTTGNQVFTGIFTAEMVLKLIALDP |
| XP_006636602.1 Na_v_1.2 II *Lepisosteus oculatus* | VDLAITICIVLNTLFMAMEHYPM-----TKEFNHVLSVGNLVFTGIFTAEMFFKVIAMDP |
| XP_006003661.1 Na_v_1.2 II *Latimeria chalumnae* | VDLAIIICIVLNTLFMAMEHYPM-----TENFSSVLSVGNLVFTGIFTAEMCFKIIALDP |
| XP_008113359.1 Na_v_1.2 II *Anolis carolinensis* | VDLAITICIVLNTLFMAMEHYPM-----TPQFNNVLSVGNLVFTGIFTAEMFLKLIAMDP |
| XP_006160314.1 Na_v_1.3 II *Tupaia chinensis* | VDLAITICIVLNTLFMAMEHYPM-----TDQFSSVLNVGNLVFTGIFTAEMILKIIAMDP |
| XP_001367154.1 Na_v_1.3 II *Monodelphis domestica* | VDLAITICIVLNTLFMAMEHYPM-----TQQFNSVLSVGNLVFTGIFTAEMVLKIIAMDP |
| XP_004601166.1 Na_v_1.3 II *Sorex araneus* | VDLAITICIVLNTLFMAMEHYPM-----TGQFSRVLTVGNLVFTGIFTAEMVLKIIAMDP |
| XP_009674477.1 Na_v_1.2 II *S. camelus australis* | VDLAITICIVLNTLFMAMEHYPM-----TEQFSSVLSVGNLVFTGIFTAEMVLKIIAMDP |
| XP_006137390.1 Na_v_1.3 II *Pelodiscus sinensis* | VDLAITICIVLNTLFMAMEHYPM-----TEQFSSVLTVGNLVFTGIFTAEMVLKIIAMDP |
| XP_008997034.1 Na_v_1.3 II *Callithrix jacchus* | VDLAITICIVLNTLFMAMEHYPM-----TEQFSSVLTVGNLVFTGIFTAEMVLKIIAMDP |
| XP_008136988.1 Na_v_1.1 II *Eptesicus fuscus* | VDLTITICIVLNTLFMAMEHYPM-----TEQFNHVLTVGNLVFTGIFTAEMFLKIIAMDP |
| XP_003795205.1 Na_v_1.1 II *Otolemur garnettii* | VDLAITICIVLNTLFMAMEHYPM-----TEHFNNVLAVGNLVFTGIFTAEMFLKIIAMDP |
| XP_005430143.1 Na_v_1.6 II *Geospiza fortis* | VDLAITICIVLNTLFMAMEHHPM-----TPEFEHVLSVGNLVFTGIFTAEMFLKLIAMDP |
| XP_005986260.1 Na_v_1.6 II *Latimeria chalumnae* | VDLAITICIVLNTLFMAMEHHPM-----TPQFEHVLLVGNFVFTGIFTAEMFLKLVAMDP |
| DAA34919.1 Na_v_1.6 II *Xenopus tropicalis* | VDLTITICIVLNTLFMAMEHYPM-----TPHFENVLVVGNLVFTGIFTAEMFLKLIAMDP |
| XP_009639044.1 Na_v_1.2 II *Egretta garzetta* | VDLAITVCIILNTLFMAMEHYPM-----TTEFNNVLKIGNQVFTGIFAAEMVLKIIAMHP |
| XP_009553849.1 Na_v_1.2 II *Cuculus canorus* | VDLAITVCIILNTLFMAMEHYPM-----TKEFNNVLKIGNQVFTGIFAAEMVLKIIAMHP |
| XP_009994594.1 Na_v_1.2 II *Chaetura pelagica* | VDLAVTVCIILNTLFMAMEHYPM-----TDDFSTALKIGNQVFTGIFAAEMVLKIIAMHP |
| XP_009892890.1 Na_v_1.2 II *Charadrius vociferus* | VDLAITVCIILNTLFMAMEHYPM-----TTNFNNVLKIGNQVFTGIFAAEMVLKIIAMHP |
| XP_010121454.1 Na_v_1.2 II *Chlamydotis macqueenii* | VDLAITLCIILNTLFMAMEHYPM-----TDNFSNVLKIGNQVFTGIFAAEMVLKIIAMHP |
| XP_005519819.1 Na_v_1.2 II *Pseudopodoces humilis* | VDLAITVCIVINTLFMAMEHYPM-----TDHFYTVLTVGNLVFTGIFAAEMVLKIIAMHP |
| XP_009086199.1 Na_v_1.2 II *Serinus canaria* | VDLAVTVCIVLNTLFMAMEHFPM-----TDHFATVLTVGNLVFTGIFAAEMVLKIIAMNP |
| XP_005946612.1 Na_v_1.5 II *Haplochromis burtoni* | LDLAITVCIVLNTLFMAMEHYPM-----TDEFNGMLTIGNLVFSGIFTAEMVLKIIALDP |
| ABA54922.1 Na_v_1.5 II *Danio rerio* | LDLAITICIVLNTLFMALEHYPM-----TDEFNSMLSIGNLVFTGIFTAEMVLKIFALDP |
| XP_007233940.1 Na_v_1.2 II *Astyanax mexicanus* | LDLAITICIVLNTLFMALEHYPM-----TDEFNKMLSVGNLVFTGIFTAEMVLKIIALDP |
| XP_005475994.1 Na_v_1.4 II *Oreochromis niloticus* | LDLGITICIVLNTLFMALEHYPM-----TDEFNTMLSVGNLVFTGIFTAEMVLKLIAMDP |
| XP_005813731.1 Na_v_1.4 II *Xiphophorus maculatus* | LDLGITICIVLNTLFMALEHYPM-----TDEFNTMLSVGNLVFTGIFTAEMVLKLVALDP |
| XP_003795197.1 Na_v_1.7 II *Otolemur garnettii* | VDLAITICIVLNTLFMAMEHHPM-----TKEFENVLAVGNLVFTGIFAAEMVLKLIAMDP |
| XP_006879389.1 Na_v_1.7 II *Elephantulus edwardii* | VDLAITICIVLNTLFMAMEHHPM-----TEEFKNMLIVGNLVFTGIFAAEMVLKLIAMDP |
| AAA89159.1 Na_v_1 II *Oryctolagus cuniculus* | VDLAITICIVLNTLFMAMEHHPM-----TEEFKNVLVVGNLVFTGIFAAEMVLKLIAMDP |
| XP_004577152.1 Na_v_1.7 II *Ochotona princeps* | VDLAITICIVLNTLFMAMEHHPM-----TEEFKNVLIVGNLVFTGIFAAEMVLKLIAMDP |
| XP_006921216.1 Na_v_1.7 II *Pteropus alecto* | VDLAITICIVLNTLFMAMEHHPM-----TEEFKSVLVVGNLVFTGIFAAEMVLKLIAMDP |
| ELR47486.1 Na_v_1.7 II *Bos mutus* | VDLAITICIVLNTLFMAMEHHPM-----TEEFKNVLVVGNLVFTGIFAAEMVLKLIAMDP |
| XP_009441903.1 Na_v_1.7 II *Pan troglodytes* | VDLAITICIVLNTLFMAMEHHPM-----TEEFKNVLAIGNLVFTGIFAAEMVLKLIAMDP |
| XP_004674637.1 Na_v_1.7 II *Condylura cristata* | VDLAITICIVLNTLFMAMEHHPM-----TDEFKDVLTVGNLVFTGIFAAEMVLKLIAMDP |
| XP_007945734.1 Na_v_1.7 II *Orycteropus afer afer* | VDLAITICIVLNTLFMAMEHHPM-----TDEFKNVLTVGNLVFTGIFAAEMVLKLIAMDP |
| XP_006180752.1 Na_v_1.7 II *Camelus ferus* | VDLAITICIVLNTLFMAMEHHPM-----TDEFKNVLTVGNLVFTGIFAAEMVLKLIAMDP |
| XP_004322655.1 Na_v_1.7 II *Tursiops truncatus* | VDLAITICIVLNTLFMAMEHHPM-----TDEFKNVLTVGNLVFTGIFAAEMVLKLIAMDP |
| XP_008829101.1 Na_v_1.7 II *Nannospalax galili* | VDLAITICIVLNTLFMAMEHHPM-----TDEFKNVLAVGNLIFTGIFAAEMVLKLIAMDP |
| ABB29444.1 Na_v_1.4 II *Tetraodon nigroviridis* | VDLGITICIILNTIFMAMEHYPM-----SADFEELLSVGNLVFTGIFTCEMVLKILAMDP |
| ABB29442.1 Na_v_1.4 II *Takifugu rubripes* | -DLGITICIILNTVFMAMEHYPM-----SADFEELLSVGNLVFTGIFTGEMVFKILAMDP |
| XP_006003324.1 Na_v_1.4 II *Latimeria chalumnae* | VDLGITICIILNTMFMAMEHYPM-----TPQFENVLSIGNLVFTGIFTAEMVFKIIALDP |
| XP_002933087.2 Na_v_1.4 II *Xenopus tropicalis* | VDLGITICIVLNTLFMAMEHYPM-----TEQFEGVLNVGNLVFTGIFAAEMFFKIIALDP |
| XP_007241580.1 Na_v_1.4 II *Astyanax mexicanus* | VDLGITICIVLNTVFMAMEHYPM-----TAEFESVLSVGNLVFTGIFTAEMVLKLIAMDP |
| AAW68223.1 Na_v_1.4 II *Thamnophis sirtalis* | VDLGITICIVLNTVFMAMEHYPM-----TEEFNNVLNVGNLVFTGIFTAEMVLKLIALDP |
| XP_007424896.1 Na_v_1.4 II *Python bivittatus* | VDLGITICIVLNTVFMAMEHYPM-----TEEFSSVLNVGNLVFTGIFTAEMVLKLIALDP |
| XP_008111415.1 Na_v_1.4 II *Anolis carolinensis* | VDLGITICIVLNTVFMAMEHYPM-----TEEFNNVLTVGNLVFTGIFTAEMVLKLIALDP |
| XP_003768502.1 Na_v_1.4 II *Sarcophilus harrisii* | VDLGITICIVLNTVFMAMEHYPM-----TPEFEHVLTVGNLVFTGIFTAEMVLKLIAMDP |
| XP_010018959.1 Na_v_1.4 II *Nestor notabilis* | VDLGITICIVLNTVFMAMEHYPM-----TEEFENVLSVGNLVFTGIFMAEMVLKLIALDP |
| KFP55758.1 Na_v_1.2 II *Cathartes aura* | VDLGITICIVLNTVFMAMEHYPM-----TEEFENVLTVGNLVFTGIFTAEMVLKLIALDP |
| KFQ96635.1 Na_v_1.2 II *Nipponia nippon* | VDLGITICIVLNTVFMAMEHYPM-----TEEFENVLTVGNLVFTGIFTAEMVLKLIALDP |
| XP_009565587.1 Na_v_1.4 II *Cuculus canorus* | VDLGITICIVLNTVFMAMEHYPM-----TEEFENVLTVGNLVFTGIFTAEMVLKLIALDP |
| KFQ13298.1 Na_v_1.5 II *Leptosomus discolor* | VDLGITICIVLNTVFMAMEHYPM-----TEEFENVLTVGNLVFTGIFTAEMVLKLIALDP |
| XP_007628932.1 Na_v_1.4 II *Cricetulus griseus* | VDLGITICIVLNTLFMAMEHYPM-----TEHFDNVLTVGNLVFTGIFTAEMVLKLIAMDP |
| XP_009073953.1 Na_v_1.4 II *Acanthisitta chloris* | VDLGITICIVLNTLFMAMEHYPM-----TEEFENVLSVGNLVFTGIFTAEMVLKLIAMDP |
| XP_008931935.1 Na_v_1.4 II *Manacus vitellinus* | VDLGITICIVLNTLFMAMEHYPM-----TEEFENVLSVGNLVFTGIFTAEMVLKLIALDP |
| XP_010226082.1 Na_v_1.4 II *Tinamus guttatus* | VDLGITICIVLNTLFMAMEHYPM-----TEEFENVLSVGNLVFTGIFTAEMVLKLIALDP |
| XP_005283172.1 Na_v_1.4 II *Chrysemys picta bellii* | VDLGITICIVLNTLFMAMEHYPM-----TEEFENVLTVGNLVFTGIFTAEMVLKLIALDP |
| XP_006274845.1 Na_v_1.4 II *A. mississippiensis* | VDLGITICIVLNTLFMAMEHYPM-----TEEFENVLTVGNLVFTGIFTAEMVLKLIALDP |
| XP_009683652.1 Na_v_1.4 II *S. camelus australis* | VDLGITICIVLNTLFMAMEHYPM-----TEEFENVLTVGNLVFTGIFTAEMVLKLIALDP |
| XP_010131382.1 Na_v_1.4 II *B. rhinoceros silvestris* | VDLGITICIVLNTLFMAMEHYPM-----TEEFENVLSVGNLVFTGIFTAEMVLKLIALDP |
| XP_009997626.1 Na_v_1.4 II *Chaetura pelagica* | VDLGITICIVLNTLFMAMEHYPM-----TEEFENVLSVGNLVFTGIFTAEMVLKLIALDP |
| XP_009700369.1 Na_v_1.4 II *Cariama cristata* | VDLGITICIVLNTLFMAMEHYPM-----TEEFENVLSVGNLVFTGIFTAEMVLKLIALDP |
| KFO60281.1 Na_v_1.2 II *Corvus brachyrhynchos* | VDLGITICIVLNTLFMAMEHYPM-----TEEFENVLTVGNLVFTGIFTAEMVLKLIALDP |
| DAA34926.1 Na_v_1.4 II *Gallus gallus* | VDLGITICIVLNTLFMAMEHYPM-----TEEFENVLTVGNLVFTGIFTAEMVLKLIALDP |
| XP_005021806.1 Na_v_1.4 II *Anas platyrhynchos* | VDLGITICIVLNTLFMAMEHYPM-----TEEFENVLTVGNLVFTGIFTAEMVLKLIALDP |
| XP_005508265.1 Na_v_1.4 II *Columba livia* | VDLGITICIVLNTLFMAMEHYPM-----TEEFENVLTVGNLVFTGIFTAEMVLKLIALDP |
| KFP89920.1 Na_v_1.2 II *Apaloderma vittatum* | VDLGITICIVLNTLFMAMEHYPM-----TEEFENVLTVGNLVFTGIFTAEMVLKLIALDP |

**Supplementary Figure 1.** Partial alignment of the S1 and S2 transmembrane segments of the VSD from Nav sequences. For comparison purposes, the Ciona voltage sensing phosphatase sequence is at the top (S1 in red, S2 in green). Asterisks denote positions mutated in this study. The four VSDs found in Nav sequences have been separated and designated I-IV..
